# Extracellular vesicle proteomics uncovers energy metabolism, complement system, and endoplasmic reticulum stress response dysregulation postexercise in males with myalgic encephalomyelitis/chronic fatigue syndrome

**DOI:** 10.1002/ctm2.70346

**Published:** 2025-06-04

**Authors:** Katherine A. Glass, Ludovic Giloteaux, Sheng Zhang, Maureen R. Hanson

**Affiliations:** ^1^ Department of Molecular Biology and Genetics Cornell University Ithaca New York USA; ^2^ Proteomics and Metabolomics Facility Institute of Biotechnology Cornell University Ithaca New York USA

**Keywords:** chronic fatigue syndrome, complement, exercise, extracellular vesicle cargo, ME/CFS, myalgic encephalomyelitis, proteomics

## Abstract

**Background:**

Myalgic encephalomyelitis/chronic fatigue syndrome (ME/CFS) is a debilitating illness characterized by post‐exertional malaise (PEM), a worsening of symptoms following exertion. The biological mechanisms underlying PEM remain unclear. Extracellular vesicles (EVs) play a key role in cell–cell communication and may provide insight into ME/CFS pathophysiology post‐exertion. Emerging evidence suggests similarities between ME/CFS and Long COVID, including PEM and overlapping immune and metabolic dysfunctions, highlighting the need for deeper mechanistic understanding.

**Methods:**

This study explores the EV proteome response to exercise in 10 males with ME/CFS and 12 well‐matched sedentary male controls. Participants underwent a maximal cardiopulmonary exercise test, and plasma samples were collected at baseline, 15 min, and 24 h postexercise. EVs were isolated from plasma using size‐exclusion chromatography and characterized with nanoparticle tracking analysis. EV protein abundance was quantified with untargeted proteomics (nanoLC‐MS/MS). Comprehensive analyses included differential abundance, pathway enrichment, protein–protein interaction networks, and correlations between EV protein dynamics and clinical or exercise physiology data.

**Results:**

ME/CFS patients exhibited many significantly altered EV proteomic responses compared with controls, including downregulation of TCA cycle‐related proteins and upregulation of complement system proteins at 15 min postexercise. Changes in proteins involved in protein folding and the endoplasmic reticulum (ER) stress response during recovery were highly correlated with PEM severity, highlighting their potential as therapeutic targets. EV protein changes postexercise were also associated with disease severity and unrefreshing sleep. Correlations between EV protein levels and the exercise parameters VO₂ peak and ventilatory anaerobic threshold were observed in controls but were absent in ME/CFS patients, suggesting disrupted EV‐mediated physiological processes.

**Conclusions:**

ME/CFS patients exhibit a maladaptive EV proteomic response to exercise, characterized by metabolic impairments, immune overactivation, and ER stress response dysregulation. These findings provide insight into the molecular basis of PEM and suggest promising targets for improving recovery and energy metabolism in ME/CFS.

**Key points:**

- EVs were isolated from plasma of ME/CFS patients and healthy controls at baseline, and 15 min and 24 h postexercise.
- Untargeted proteomics revealed dysregulation in energy metabolism, the complement system, and the endoplasmic reticulum stress response.
- Changes in EV protein levels postexercise are associated with post‐exertional malaise.
- These findings suggest promising therapeutic targets for post‐exertional malaise and ME/CFS pathophysiology.

## Graphical abstract

1. EVs were isolated from plasma of ME/CFS patients and healthy controls at baseline, and 15 min and 24 h postexercise.
2. Untargeted proteomics revealed dysregulation in energy metabolism, the complement system, and the endoplasmic reticulum stress response.
3. Changes in EV protein levels postexercise are associated with post‐exertional malaise.
4. These findings suggest promising therapeutic targets for post‐exertional malaise and ME/CFS pathophysiology.

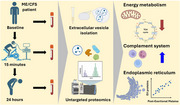

## 1 BACKGROUND

Myalgic encephalomyelitis/chronic fatigue syndrome (ME/CFS) is a chronic, systemic illness affecting millions worldwide, characterized by a broad spectrum of symptoms including persistent and disabling fatigue, cognitive impairments, pain, and sleep disturbances.^1^ The hallmark symptom of ME/CFS, post‐exertional malaise (PEM), distinguishes it from other fatigue‐related conditions.^2^, ^3^, ^4^, ^5^ PEM involves a severe exacerbation of symptoms that often starts 12–24 h following even minimal physical or mental exertion, and leaves patients debilitated for days or longer after activity.^6^ Despite decades of research, the exact aetiology of ME/CFS remains elusive, though growing evidence suggests a complex interplay between immune dysregulation, metabolic dysfunction, and cellular abnormalities.^7^, ^8^, ^9^

While physical exercise is generally beneficial for overall health, improving cardiovascular and muscular function in healthy individuals,^10^, ^11^, ^12^ it has paradoxical effects in ME/CFS patients, who often suffer from exercise intolerance. Studies using cardiopulmonary exercise testing (CPET) have shown that ME/CFS patients exhibit abnormal physiological responses to exertion, including reduced aerobic capacity, impaired oxygen utilization, and metabolic dysregulation.^13^, ^14^, ^15^, ^16^ These findings suggest that, unlike healthy individuals, ME/CFS patients experience an inability to recover from exertion, leading to debilitating PEM. However, the biological mechanisms underlying these responses remain poorly understood.

One emerging avenue of research involves the study of extracellular vesicles (EVs): small, membrane‐bound particles released by cells that play critical roles in intercellular communication. EVs carry a wide variety of molecular cargo, including proteins, RNA, and lipids, and can mediate diverse biological processes such as immune modulation, metabolism, and tissue repair.^17^, ^18^, ^19^ Exercise has been shown to increase the release of EVs into circulation, with their cargo reflecting the body's response to exercise‐induced stress.^20^, ^21^ In healthy individuals, EVs released after exercise have been implicated in enhancing metabolic adaptation, inflammation resolution, and tissue repair.^22^, ^23^, ^24^, ^25^

Several studies have explored the role of EVs in ME/CFS, providing preliminary insights into how EV signalling may be disrupted in this condition. Castro‐Marrero et al.^26^ were among the first to analyze EVs in ME/CFS patients, finding an increased concentration of smaller EVs in patients compared with controls, suggesting potential differences in EV production or clearance. Almenar‐Pérez et al.^27^ expanded on these findings by showing dysregulation in EV‐associated neuroimmune pathways, further supporting the idea that EVs may contribute to immune dysfunction in ME/CFS. Additionally, our group has conducted several studies on the content of EVs in ME/CFS patients, including cytokines, revealing a higher concentration of EVs in the patient group and correlations between EV‐associated cytokines and symptom severity.^28^, ^29^, ^30^ Eguchi et al.^31^ used untargeted proteomics to analyze the protein content of EVs from a subset of ME/CFS patients and controls, identifying a distinct protein profile in ME/CFS patients, including actin network proteins and 14‐3‐3 family proteins, further suggesting that EVs carry unique molecular cargo in this condition. While these studies have highlighted the potential role of EVs in ME/CFS, much remains unknown about the specific molecular cargo of EVs in this condition and during PEM.

Our group recently performed the first comprehensive proteomic analysis of EVs in female ME/CFS patients before and after exercise.^28^ We identified significant changes in proteins involved in immune regulation, brain signalling (including the 14‐3‐3 family proteins), coagulation processes, and dysregulation of smooth and skeletal muscle proteins, all of which may contribute to the heightened immune responses and metabolic dysregulation observed in these patients during PEM.^28^ Since the majority of ME/CFS patients are female, with an approximate female‐to‐male ratio of 2:1,^32^ there is a lack of research focusing on male ME/CFS patients, particularly in EVs. Given the growing recognition of sex differences in immune function and disease pathology,^33^, ^34^ including in ME/CFS studies of immunity and metabolism^13^, ^35^, ^36^, ^37^ it is essential to comprehensively investigate alterations in EV signalling in male patients versus male controls in addition to studying females.

The current study addresses this gap by focusing on male ME/CFS patients. We analyzed the proteomic cargo of EVs isolated from the plasma of 10 male ME/CFS patients and 12 age‐ and BMI‐matched healthy sedentary male controls before, 15 min, and 24 h after a maximal exercise challenge. By using nanoparticle tracking analysis (NTA) to measure EV size and concentration and quantitative proteomics to assess EV protein content, we aimed to better understand mechanisms related to EV signalling in ME/CFS generally and in the context of PEM. Given the limited research on EVs in ME/CFS, particularly in males, our findings could provide valuable insights into the biological underpinnings of PEM and the broader disease process in male patients. We hypothesize that similar to females, male ME/CFS patients will exhibit dysregulation in EV proteomic cargo, particularly in the acute response to exercise, and that characterizing this altered signalling will generate new perspectives on sex‐specific disease mechanisms.

## 2 MATERIALS AND METHODS

### 2.1 Population characteristics

This study included a cohort of 10 males diagnosed with ME/CFS and 12 age‐ and BMI‐matched male healthy sedentary controls (a subset of a larger study, ClinicalTrials.gov Identifier: NCT04026425). All ME/CFS cases were diagnosed by expert physicians according to the 2003 Canadian consensus criteria.^38^ Participants in both groups were excluded if they were diabetic, smoked cigarettes, consumed excessive amounts of alcohol, had an orthopaedic limitation preventing them from performing the cardiopulmonary exercise test (CPET), or had any of the following diagnoses: an autoimmune disease, schizophrenia, major depressive disorder, bipolar disorder, or an anxiety disorder. Healthy subjects included in the present study were categorized as “low‐active”: they had a sedentary job and no regular organized physical activity in the past 6 months. All subject participation was completed prior to the onset of the COVID‐19 pandemic in March 2020.

### 2.2 Cardiopulmonary exercise testing, surveys, and blood collection

All subjects performed a maximal CPET with the following protocol: stationary cycling with a workload increase of 15 watts per minute until volitional exhaustion, ensuring a respiratory exchange ratio greater than 1.1, which indicates maximum effort. Further technical details on the CPET protocol can be found elsewhere.^15^, ^16^

Blood samples were collected at three time points: prior to the CPET (0 h), 15 min post‐CPET (15 min), and 24 h post‐CPET (24 h). Peripheral blood was drawn into K2 EDTA (K2E) Plus Blood Collection Tubes (BD Vacutainer) and centrifuged to pellet red blood cells. The resulting plasma samples were stored at −80°C until processing.

Recent health and functional status were assessed using several tools, including the Short Form 36 Health Survey (SF‐36v2 Health Survey),^39^ the multidimensional fatigue inventory (MFI) scale,^40^ the Bell activity scale score^41^ and custom questionnaires. Additionally, clinical symptom severity was evaluated at various time points throughout the study, including both 0 and 24 h, using a modified version of the chronic fatigue syndrome specific symptom severity (SSS) score.^42^

### 2.3 Extracellular vesicles purification and characterization

EVs were isolated from plasma samples using size exclusion chromatography with Izon qEV original columns (Izon Science) following the manufacturer's instructions. Before proteomic analysis, Halt Protease Inhibitor Cocktail (1X) (Thermo Scientific) was added to all samples.

NTA to determine the concentration and size distribution of isolated EVs was performed with the NanoSight NS300 (Malvern). After thawing, samples were diluted 1:2000 in 1× PBS, and 1 mL was injected through the laser chamber (NanoSight Technology). For each sample, three 60‐second digital videos were recorded and analyzed by the NanoSight NTA 2.3 software to determine the size and concentration of nanoparticles. The mean results from the three recordings are reported. To evaluate differences in size and EV concentration between cases and controls, the Wilcoxon rank sum test was applied (*p* < .05). The Wilcoxon signed‐rank test was used to compare size and concentration at different time points within groups (*p* < .05).

EV suspensions were visualized by TEM. Undiluted samples were placed onto copper formvar carbon‐coated TEM grids (Electron Microscopy Sciences), allowed to adsorb for 5 min, and then washed twice in water for 30 s. The samples were negatively stained by floating the grids on a drop of 2% uranyl acetate for 30 s. After staining, the grids were blot‐dried with Whatman paper and imaged with a JEOL JEM 1230 Transmission Electron microscope (JEOL USA, Inc.).

### 2.4 Proteomics

Detailed information can be found in Giloteaux et al.^28^ In brief, a TMT 10‐plex shotgun proteomics analysis was performed. Nine protein‐extracted EV samples from three subjects at 0 h, 15 min, and 24 h postexercise were included in each of two TMT 10‐plex sets, one for the ME/CFS group and one for the control group. The remaining channel in each TMT set was used for a pooled reference of proteins from all 18 samples to bridge the results between groups. A total of four TMT experiments were conducted (Figure S1).

For each sample, 15 µg of protein was reduced, alkylated, and digested with trypsin using an S‐Trap Micro Spin column (Protifi). The resulting tryptic peptides were labelled individually according to Thermo Scientific's TMT protocol and pooled together in each set, and 80 µg of labelled peptides were fractionated using a Pierce High pH reversed‐phase peptide fractionation kit (Thermo‐Fisher Scientific), and the resulting three fractions for each set were analyzed by nanoLC‐MS/MS with an Orbitrap Eclipse mass spectrometer equipped with a nanospray Flex Ion Source (Thermo‐Fisher Scientific) and coupled with the UltiMate3000 RSLCnano (Dionex). All raw files were acquired under the Real Time Research SPS MS^3^ method^43^ and processed with the Thermo Scientific Xcalibur software 4.3.

Raw MS spectra were processed using the Sequest HT search engine within Proteome Discoverer 2.3 software, searching against the Homo Sapiens NCBI database (downloaded 5/5/2022, 81785 sequences). Various dynamic and static modifications were specified, and the TMT quantification method was used to calculate reporter ion abundances. Signal‐to‐noise (S/N) values were used for quantitation, with normalization on the total peptide amount for each sample. Protein expression across different TMT experiments was assessed by comparing sample ratios to the pooled reference.^44^

### 2.5 Data processing and statistical analysis

A total of 1594 proteins were identified across the four TMT experiments (Table S2). Prior to analysis, lipoproteins and keratins, common contaminants in EV proteomics studies^45^ were excluded. Proteins were included in the final dataset if they were detected in at least 75% of the TMT experiments (i.e., in at least 3 out of the 4 TMT experiments) and 75% of all samples (group‐agnostic). Proteins detected in fewer than 3 TMT experiments (<75%) or fewer than 75% of all samples were excluded from analysis. Detection frequencies for each analyzed protein ME/CFS and control samples are provided in Table S2. Missing values for 865 proteins that met these criteria were imputed using the random forest method (*missForest* R package, default parameters).^46^, ^47^ The imputation included all proteins along with categorical variables without missing data: experimental group (ME/CFS vs. Control), time point (0 h, 15 min and 24 h) and TMT experiment (1‐4).

Original and imputed data for 865 EV proteins were analyzed at all three time points. Due to the experimental design, comparisons between ME/CFS and control samples could only be made within each TMT experiment. To statistically compare the groups at each time point or to assess within‐subject fold changes over time (15 min vs. 0 h, 24 h vs. 0 h, and 24 h vs. 15 min) between ME/CFS and control groups, a bootstrapping approach was used. For each protein, the following algorithm was applied to generate 10,000 bootstrapped datasets from all possible combinations of ME/CFS:control subject fold changes (nine combinations for TMT experiments with three ME/CFS patients and three controls, and six combinations for TMT experiments with two ME/CFS patients and three controls): (1) for each TMT experiment, randomly select with replacement one‐third of the possible fold changes (three for experiments with three ME/CFS patients, and two for those with two ME/CFS patients); (2) calculate the median of the fold changes, which is more robust to outliers than the mean. To compare ME/CFS with the control group, 95% confidence intervals were calculated for the 10,000 bootstrapped medians. The null hypothesis assumed that the ratio of the two groups is 1 (indicating no difference). Confidence intervals were adjusted for false discovery using the BY procedure (*q* < .1).^48^ Protein levels are considered significantly different between groups if the adjusted confidence interval did not include 1. A confidence interval below 1 indicates that the protein level is significantly lower in ME/CFS compared with controls, while a confidence interval above 1 indicates a significantly higher level in the ME/CFS group.

To compare changes in protein levels over time within the ME/CFS and control groups, we calculated the within‐subject fold changes for 15 min versus 0 h, 24 h versus 0 h, and 24 h versus 15 min. Since all three samples for each subject were measured within the same TMT experiment, bootstrapping was not required for this analysis. For each protein, the mean fold changes were compared with 1 using a *t*‐test, with the null hypothesis being that the mean was equal to 1 (no difference). *p*‐values were adjusted using the Benjamini and Hochberg false discovery rate (BH FDR) correction procedure (*q* < .1).^49^

### 2.6 Pathway analysis

Significantly different EV proteins were subjected to functional annotation using Reactome pathway enrichment analysis with Enrichr software available online (https://maayanlab.cloud/Enrichr/).^50^ The *p*‐value is determined using Fisher's exact test, assuming independence of genes and the *q*‐value, an adjusted *p*‐value, is computed using the BH method. Reactome terms and pathways with an adjusted *p*‐value FDR < .05 were considered significantly enriched.

GSEA was performed using the bootstrapped median Log2 fold change between groups as the ranking metric implemented through the *multiGSEA* R package.^51^ *multiGSEA* is a wrapper for *fgseaMultilevel* (with the following parameters: eps = 0, minSize = 5, maxSize = 500, nPermSimple = 10,000).^52^ Significance is determined by comparing a gene set to many random permutations of gene sets of the same size, followed by BH FDR correction (*q* < .05). Entrez gene symbols were used for IDs, and the following databases were queried on 26 July 2024: KEGG, Reactome, Wikipathways, and Panther GO. The *graphite* Bioconductor R package, integrated within *multiGSEA*, was used to import the latest versions of these databases from their respective URLs.^53^

### 2.7 Correlation with clinical parameters

This analysis was done separately for ME/CFS and control cohorts. To investigate the association between postexercise changes in protein levels and ME/CFS symptoms, exercise physiology, and demographic parameters, Spearman correlation coefficients were calculated for within‐subject fold changes over time (15 min/0 h, 24 h/0 h, and 24 h/15 min) for each protein against various clinical parameters. Significance was assessed by generating bootstrapped 95% confidence intervals for each correlation coefficient and calculating associated *p*‐values using the *bootcorci* package, with the null hypothesis that the coefficient equals 0. A correlation was considered significant if the 95% confidence interval did not include 0. For each clinical parameter, *p*‐values were adjusted using BH FDR correction (*q* < .1). Significant and strong correlations are defined as those with an absolute value of Spearman *R* > .8 and *q *< .1.

Demographic parameters included age, BMI, and for the ME/CFS group, duration of illness. Additionally, survey data that reflect physical function and disease severity were also evaluated, including the Bell Activity Scale score (ranging from 10 to 100, with 100 indicating greater activity), the SF‐36 Physical Component Score (ranging approximately from 11 to 64, with higher scores indicating better physical function), the SF‐36 General Health score, and the proportion of waking time spent in a reclined position.

We also examined symptom severity. For the SSS, each subject in both the ME/CFS and control groups rated their symptoms on a scale from 0 to 10 (0 = not present and 10 = very high) at three different times: (1) on average over the past month, (2) on the morning of the CPET (0 h) and (3) 24 h after the CPET (24 h). The ΔSSS are calculated by subtracting the score at 0 h from the score at 24 h. Therefore, a ΔSSS below 0 indicates an improvement in that symptom following exercise, while a ΔSSS above 0 suggests that the symptom worsened 24 h postexercise. The following symptoms were included in our analysis: fatigue, impaired memory or concentration, recurrent sore throat, lymph node tenderness, muscle tenderness or pain (myalgia), joint pain (arthralgia), headache, unrefreshing sleep, and PEM. The Multidimensional Fatigue Inventory‐20 total score was used as another metric of general fatigue, but only in ME/CFS subjects (range 20–100, with higher scores indicating greater fatigue).

### 2.8 Protein interaction networks

PPI networks were built using the STRING v12.0 software available online (https://string‐db.org/),^54^ which integrates diverse sources of interaction data to create comprehensive interaction maps. These networks are constructed based on multiple lines of evidence, including experimental data, computational predictions, co‐expression patterns, and information from curated databases. STRING assigns confidence scores to each interaction, with values ranging from 0 to 1, where higher scores indicate stronger and more reliable evidence supporting the interaction.

To assess the statistical significance of the observed interactions within the network, STRING calculates a PPI enrichment p‐value. This *p*‐value is determined using a hypergeometric test, which compares the observed number of interactions among a set of proteins to what would be expected by random chance. This statistical approach allows for the identification of significant interaction patterns, ensuring that the constructed networks are biologically relevant and not a result of random associations.

### 2.9 Data visualization

Unless otherwise specified, all plots were generated in R with *ggplot2*.

## 3 RESULTS

### 3.1 Study subjects and overview

Ten males with ME/CFS and 12 age‐ and BMI‐matched sedentary male controls participated in this study (Table 1). The ME/CFS group had a mean age of 46.4 years (±7.7), while the control group had a mean age of 44.5 years (±12.8), with no significant difference between the two groups (*p* = .67). Similarly, the BMI of the ME/CFS group was 29.7 (±3.6) compared with 27.0 (±3.7) in the control group, also showing no significant difference (*p* = .10). The type of onset for ME/CFS was evenly split between sudden and gradual onset, with a median disease duration of 16.5 years, ranging from 3 to 36 years. ME/CFS patients displayed significantly greater levels of disability, as reflected by a mean Bell's Disability Scale score of 38.7 (±16.7), which was substantially lower than the controls’ score of 94.2 (±6.7) (*p* = 6.00 × 10⁻⁷). Additionally, ME/CFS patients scored lower on the SF‐36 Physical Component Summary (28.7 ± 7.3 vs. 55.9 ± 6.1, *p* = 3.12 × 10⁻⁸) and Mental Component Summary (45.7 ± 6.5 vs. 55.6 ± 5.9, *p* = 1.60 × 10⁻^3^) compared with controls, indicating that the disease has a significant impact on both physical and mental health. In terms of exercise capacity, the ME/CFS group did not significantly differ from controls for VO₂ peak or ventilatory anaerobic threshold (VAT), although there was a trend towards lower VAT (*p* = .09).

**TABLE 1:** Demographic characteristics of study subjects.

	ME/CFS	Controls	*T*‐test *p*‐value
*n*	10	12	NA
Age (years)	46.4 ± 7.7	44.5 ± 12.8	.67
BMI	29.7 ± 3.6	27.0 ± 3.7	.10
Type of onset			
Sudden	50%	NA	NA
Gradual	50%	NA	NA
Years with ME/CFS^a^	16.5 (3–36)	NA	NA
Bell's disability Scale	38.7 ± 16.7	94.2 ± 6.7	6.00 × 10^−7^
SF‐36			
Physical component summary	28.7 ± 7.3	55.9 ± 6.1	3.12 × 10^−8^
Mental component summary	45.7 ± 6.5	55.6 ± 5.9	1.60 × 10^−3^
MFI total	78.9 ± 10.1	NA	NA
VO_2_ peak (mL·kg^−1^·min^−1^)	22.6 ± 4.9	24.6 ± 5.6	.40
Ventilatory anaerobic threshold (mL·kg^−1^·min^−1^)			
10.8 ± 2.9	13.2 ± 3.4	.09	

*Note*: All data are presented as mean ± SD unless otherwise specified.

^a^Years with ME/CFS are presented as the median and range in parentheses.

**FIGURE 1:**
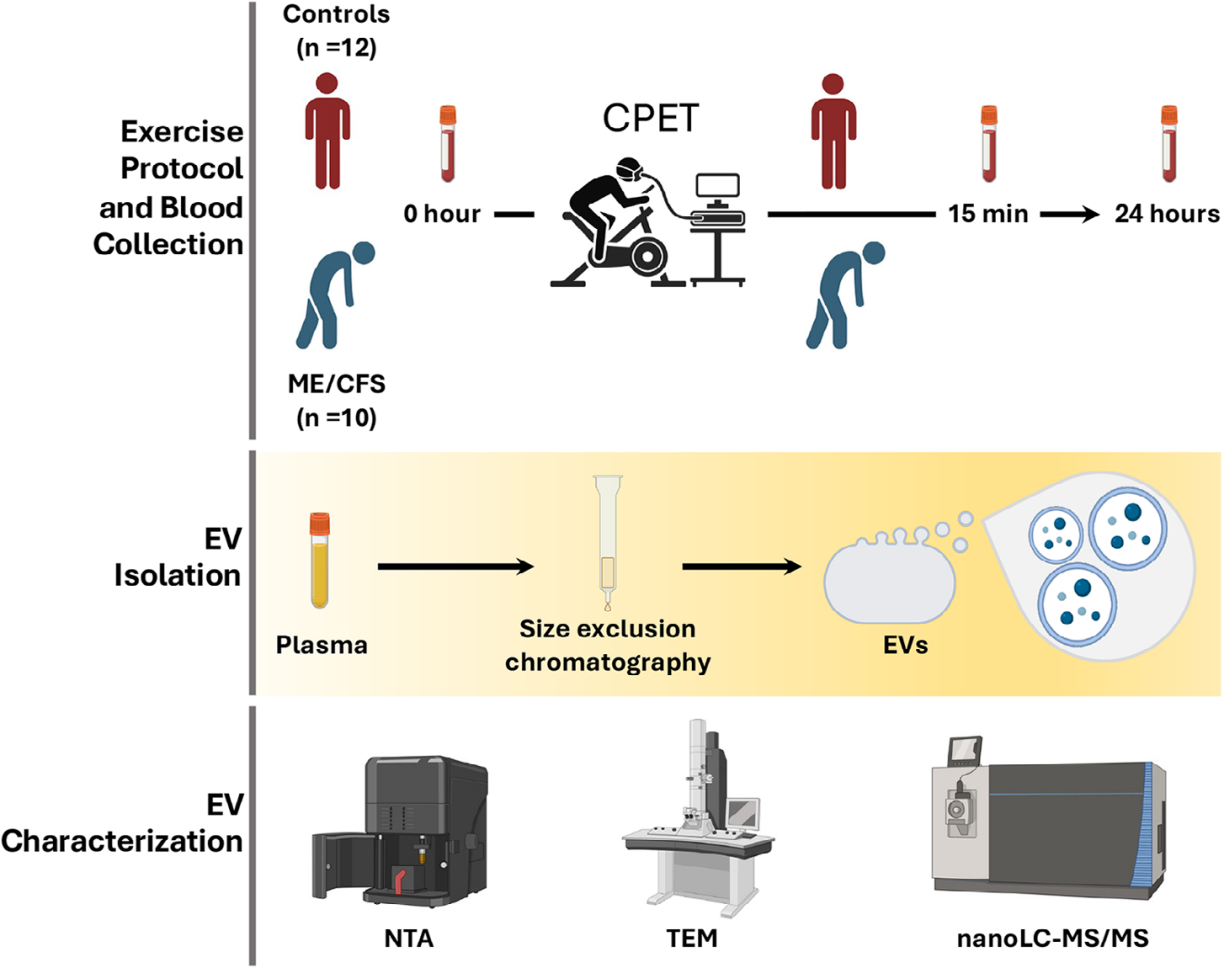
Study Design. The study included a CPET and subsequent blood sample collection at baseline (0 h), 15 min postexercise, and 24 h postexercise. After blood collection, EVs were isolated using size exclusion chromatography. Isolated EVs were characterized using NTA, TEM, and untargeted proteomics via nanoLC‐MS/MS.

The study design is illustrated in Figure 1. All participants underwent a maximal CPET on a stationary cycle ergometer. Blood samples were taken at three time points: baseline (0 h), 15 min post‐CPET, and 24 h post‐CPET. EVs were isolated from plasma using size exclusion chromatography. A subset was imaged using transmission electron microscopy (TEM), and EV size, distribution, and concentration were assessed via NTA. EV protein cargo was quantified through untargeted proteomics using nanoLC‐MS/MS.

### 3.2 Characterization of EVs

NTA revealed that the mean sizes of particles were not statistically different between the ME/CFS and control groups at any time point (see Figure S3; Table S1).

**FIGURE 2:**
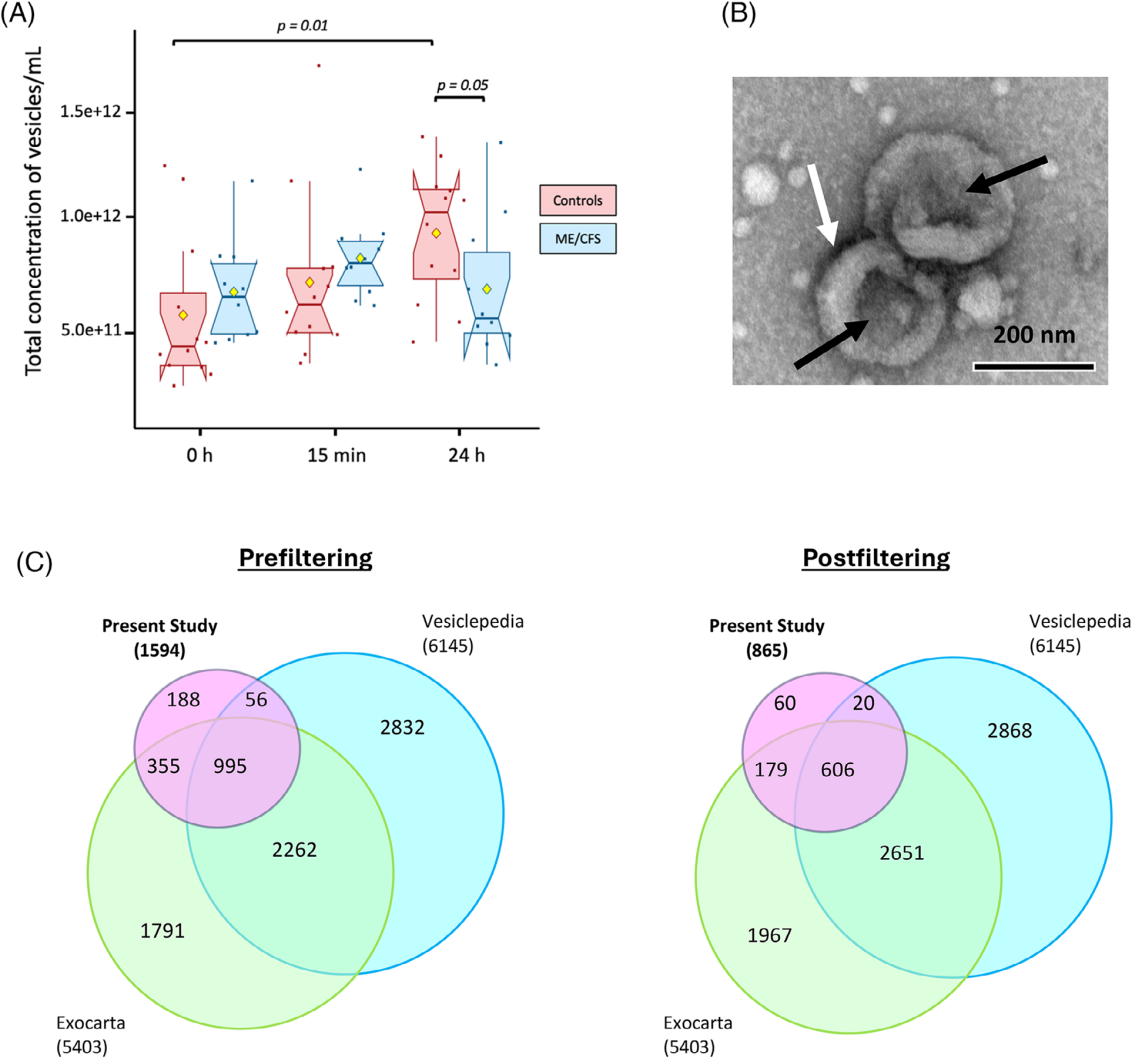
Nanoparticle tracking analysis and transmission electron microscopy. (A) Vesicle concentration: The total concentration of vesicles per mL of plasma was measured in both ME/CFS subjects and healthy controls. The yellow dot indicates the mean concentration. The nonparametric Wilcoxon rank sum test was used to determine the significance of differences between the two groups, while the Wilcoxon signed‐rank test assessed differences over time within subjects (*p* < .05). (B) transmission electron microscopy: a representative TEM image obtained by negative staining shows the morphological structures of plasma‐derived EVs isolated by size exclusion chromatography. Black arrows indicate EV‐like nanoparticles and white arrows point to the characteristic lipid bilayer (scale bar: 200 nm). (C) Comparison of our dataset's proteomic coverage with manually curated vesicle databases Vesiclepedia and Exocarta for all identified EV proteins (prefiltering) and proteins remaining after filtering out those with more than 25% missing values (postfiltering).

At 0 h and 15 min postexercise, the mean concentration of vesicles per mL of plasma trended higher in the ME/CFS group compared with the control group, although these differences were not statistically significant (*p* = .12 for 0 h and *p* = .06 for 15 min respectively, Figure 2A; Table S1). However, 24 h postexercise, the control group exhibited a trend towards a higher concentration of circulating EVs compared with the ME/CFS group (*p* = .05, Figure 2A; Table S1). Lastly, the mean vesicle concentration in ME/CFS patients returned to a value close to baseline 24 h postexercise (6.2 × 10¹¹ to 6.0 × 10¹¹ vesicles/mL), while in the control group, the mean EV concentration significantly increased by 1.9‐fold at 24 h compared with baseline (*p* = .01, Figure 2A, Table S1).

Selected EVs were further characterized using TEM and showed features typical of EV morphology (Figure 2B). They appear as round structures with a slight central concavity, which gives them the appearance of a cup and a characteristic bilayered membrane, (Figure 2B, full micrograph in Figure S2). Smaller particles, likely representing lipoproteins, as well as a few larger particles ranging from 200 to 350 nm in diameter, were also observed in the samples.

The proteome of the EVs was then analyzed using untargeted nanoLC‐MS/MS with Tandem Mass Tag (TMT)‐based quantitative proteomics. In total, 1594 proteins were identified (the full list can be found in Table S2). After filtering out proteins with missing values in more than one out of four TMT experiments, 865 proteins were retained for further analysis. Of these, 575 proteins had no missing data, while the remaining missing values were imputed using random forest (RF, *missFores*t R package, normalized root mean square error .44). We chose to impute data using RF because it is recognized as the optimal method for imputation in mass spectrometry data when the cause of missingness is unknown, such as in cases of missing completely at random.^55^ RF also effectively manages non‐linear data and outliers without requiring feature scaling.^55^, ^56^

We cross‐referenced our EV protein dataset (pre‐ and postfiltered) with two well‐established EV proteome databases, Exocarta^57^ and Vesiclepedia.^58^ Impressively, 88% and 93% of the proteins in our datasets matched those found in the databases, pre‐ and postfiltering, respectively (Figure 2C). Additionally, we identified several well‐known EV markers,^45^ including tetraspanins CD9 and CD63, cytosolic TSG101, and flotillin FLOT1.

### 3.3 Differences in EV proteomic cargo in ME/CFS versus controls peak at 15 min postexercise

**FIGURE 3:**
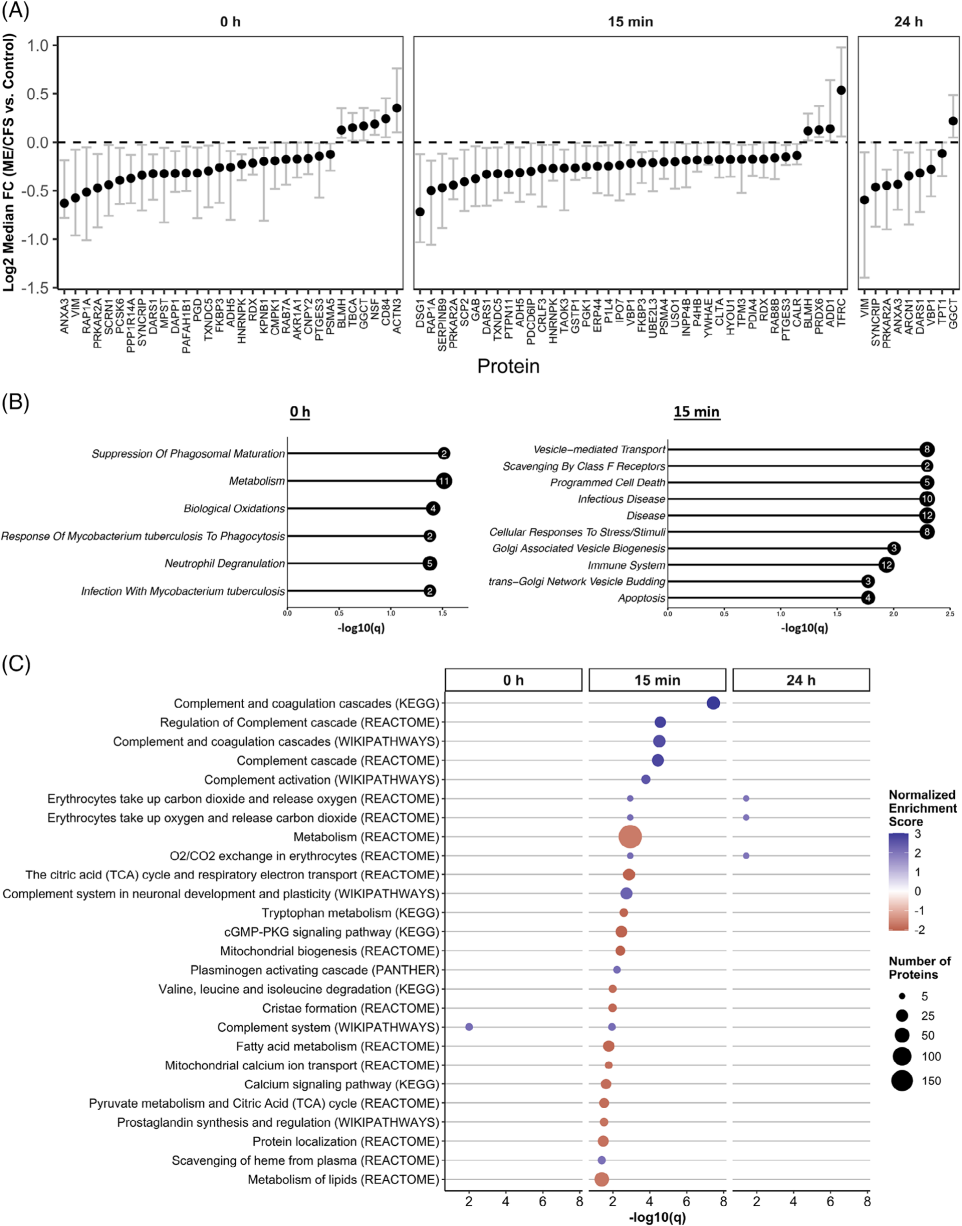
Differences in EV protein levels between ME/CFS and controls at each time point. (A) The y‐axis shows the median Log2 fold change (ME/CFS vs. controls) of 10,000 bootstrapped datasets at each time point. Grey bars with caps show the FDR‐adjusted confidence intervals (*q* < .1). A protein is significant after FDR correction (DAPs) if the adjusted CI does not include 0. (B) Bar plots showing—Log10(*q*) of the top 10 most significant Reactome pathways (FDR < .05) enriched for EV proteins that are significantly different (*q* < .05) between ME/CFS and controls at 0 h and 15 min postexercise. The number inside the bubble shows the number of EV proteins in each pathway. (C) Pathways significantly enriched in ME/CFS vs. controls by GSEA, only pathways with *q* < .05 are shown. Pathways are listed in order of significance at 15 min postexercise. Bubble size shows the number of proteins in that set, and the colour shows the normalized enrichment score (NES).

We used a bootstrapping approach to compare EV protein abundance between ME/CFS patients and control subjects. This method allowed for direct comparisons of samples within each TMT experiment (as detailed in the Methods and Figure S1). The differentially abundant proteins (DAPs) identified between ME/CFS and controls at each time point are illustrated in Figure 3A (*q* < .1, Benjamini and Yekutieli false discovery rate (BY FDR) correction procedure for confidence intervals^48^). A protein was considered differentially abundant if its adjusted confidence interval for the fold change between groups did not include 0.

We identified 31 DAPs at baseline, 39 at 15 min postexercise, and nine at 24 h postexercise. Most DAPs at all time points exhibited decreased levels of EVs from ME/CFS patients compared with controls, as indicated by the Log2 median fold change being below 0 (Figure 3A). Among these, PRKAR2A and DARS1 consistently showed decreased abundance across all three time points, suggesting persistent dysregulation in ME/CFS EVs, independent of exercise. PRKAR2A (Protein Kinase A Regulatory Subunit 2 Alpha) regulates the activity of protein kinase A (PKA) and is involved in various cellular processes including metabolism, cell division, and memory. DARS1 (Aspartyl‐tRNA Synthetase 1) catalyzes the attachment of aspartic acid to tRNA, which is essential for protein synthesis.

Notably, ANXA3, VIM, GGCT, and SYNCRIP were differentially abundant at both 0 and 24 h, which suggests any potential changes due to exercise have stabilized at 24 h. ANXA3 is linked to membrane repair, VIM to cellular integrity, GGCT to oxidative stress management, and SYNCRIP to RNA processing, suggesting persistent cellular stress and dysfunction in ME/CFS.

Additionally, eight proteins, ADH5, BLMH, FKBP3, HNRNPK, PTGES3, RAP1A, RDX, and TXNDC5, were differentially abundant at 0 h and 15 min postexercise. ADH5 and BLMH are involved in detoxification, with BLMH showing increased levels in ME/CFS EVs compared with controls, suggesting a heightened stress response or protein degradation. FKBP3 is involved in protein folding and immunoregulation, HNRNPK in mRNA processing, PTGES3 in inflammation, RAP1A in cell adhesion, and RDX in cell structure. TXNDC5 is a member of the protein disulfide isomerase (PDI) family of endoplasmic reticulum proteins that can catalyze protein folding. The consistent dysregulation of these proteins before and after exercise suggests it is a baseline characteristic of ME/CFS, unaffected by immediate exercise responses, which again points to a chronic underlying pathology.

VHL binding protein 1 (VBP1) was the sole protein differentially abundant at both postexercise time points (15 min and 24 h), showing decreased levels in ME/CFS patients, indicative of exercise‐induced dysregulation without recovery by 24 h. Significantly, 28 DAPs were unique to the 15 min postexercise time point, with 25 showing decreased levels in ME/CFS patients, highlighting a potential failure in rapid EV‐mediated signalling responses to exercise in ME/CFS.

To gain functional insight into these DAPs, we performed pathway analyses using Enrichr with the list of all DAPs at each time point as the input (Figure 3B, *q* < .05, Fisher's exact test followed by Benjamini and Hochberg false discovery rate (BH FDR) correction). At baseline, the most significantly enriched pathways, featuring the largest number of DAPs, were metabolism (11 proteins), neutrophil degranulation (5 proteins), and biological oxidations (4 proteins). This suggests that even at rest, ME/CFS patients exhibit substantial disruptions in metabolic processes and immune functions, specifically those involving neutrophils, which are key players in the body's first line of defence.

At 15 min postexercise, three of the top 10 most significant pathways are related to the immune system and disease (12 proteins). This highlights a potential failure of the appropriate EV‐mediated immune response in ME/CFS patients during the acute recovery phase after exercise. Another three of the top 10 pathways are involved in vesicle‐mediated transport and biogenesis, suggesting that the large‐scale dysregulation of EV proteomic cargo in response to exercise may be linked to defects in the production of EVs themselves. The remaining top pathways with the highest number of proteins involved (8) are related to the cellular response to stress and stimuli. The reduction in the cellular response to stress/stimuli‐related proteins in ME/CFS patients’ EVs 15 min postexercise is likely contributing to their exercise intolerance.

At 24 h postexercise, with only nine DAPs identified, there were no significantly enriched pathways that met our strict threshold. The significant pathway analysis results can be found in Tables S3 and S4 for the 0 h and 15 min time points, respectively.

We next performed a quantitative enrichment with gene set enrichment analysis (GSEA).^59^ Proteins were ranked based on the median fold‐change between ME/CFS and controls for all 865 proteins at each time point, enabling the detection of pathways that exhibit collective changes in EV protein levels, even if individual proteins do not show significant alterations. Protein set enrichment was assessed using four databases (KEGG, Reactome, Wikipathways, and PANTHER, *multiGSEA* R package,^51^ complete results in Table S5).

Our analysis revealed significant pathway alterations in ME/CFS patients compared with controls, particularly at 15 min postexercise, where 26 pathways were significantly enriched. In contrast, only one pathway was significantly dysregulated at baseline and three pathways at 24 h postexercise (Figure 3C, *q* < .05, Table S5). The top five most significant pathways, predominantly involving the complement and coagulation cascades, were consistently identified across three different databases. Core contributors to the enrichment of these pathways included key components of the complement system, such as C3, CFH, C4BPA, and the classical complement pathway proteins C1qA/B/C. For the coagulation cascade, crucial members included fibrinogen chains FGA, FGB, and FGG, as well as coagulation factors XI and V, and plasminogen. For further details, refer to Table S5, which provides the leading‐edge proteins for each set. The strong positive normalized enrichment scores (NES), ranging from 2.5 to 3, indicate elevated levels of EV proteins in male ME/CFS subjects relative to controls, with 10 to 35 proteins contributing to overactivation of these pathways.

We observed strong negative NES for many metabolism‐related pathways at 15 min, suggesting a failure in male ME/CFS patients to mount an adequate metabolic signalling response to exercise in EVs. These dysregulated pathways encompass broad metabolic processes, including general metabolism (185 proteins), the citric acid (TCA) cycle and respiratory electron transport (26 proteins), valine, leucine, and isoleucine degradation (7 proteins), tryptophan metabolism (8 proteins), fatty acid metabolism (21 proteins), pyruvate metabolism and the TCA cycle (13 proteins), and lipid metabolism (46 proteins). The consistent downregulation in these pathways points to a potential energy production and utilization deficit in ME/CFS patients, which might underlie the profound fatigue and exercise intolerance characteristic of the disease.

Moreover, three of the pathways, all with reduced protein levels in EVs as indicated by negative NES, are specifically related to mitochondrial functions: mitochondrial biogenesis (12 proteins), cristae formation (8 proteins), and mitochondrial calcium ion transport (6 proteins) (Figure 3C). Mitochondrial dysfunction could again contribute to the impaired recovery and postexertional malaise observed in these patients.

Interestingly, the only pathway significantly enriched at 0 h, the complement system (Wikipathways), remained elevated 15 min postexercise. This suggests that the complement system in ME/CFS patients is already primed for overactivation prior to exercise, and this overactivation is exacerbated during the immediate postexercise phase. This chronic activation of the complement system could be a contributing factor to the persistent inflammation and immune dysregulation observed in ME/CFS.

Finally, the pathways significantly enriched at 24 h postexercise, all involving O_2_/CO_2_ exchange in erythrocytes (comprising the same 5 core proteins HBB, HBA1, CA2, SLC4A1, and CA1), were also significantly enriched at 15 min postexercise. Thus, overactivation of this process triggered by exercise in ME/CFS patients did not return to baseline by 24 h, suggesting a prolonged disruption in gas‐exchange processes.

### 3.4 Altered postexercise temporal dynamics of EV proteomic cargo in ME/CFS subjects versus controls

To explore how the EV proteomic cargo changes over time postexercise in ME/CFS subjects compared with controls, we used the same bootstrapping statistical approach, this time focusing on the within‐subject fold changes 15 min/0 h, 24 h/0 h, and 24 h/15 min (ratios over time) between the two groups. This analysis helps us better understand the temporal dynamics of altered EV proteomic signalling in ME/CFS as induced by exercise.

We also assessed the changes in protein levels over time within each group using a Student's *t*‐test, comparing the within‐subject fold changes to 1. Bootstrapping analysis was not appropriate in this case because the three samples for each subject were included in the same TMT experiment and could therefore be directly compared. However, no significant results were found after FDR correction (BH, *q* < .1). This was not surprising considering the small sample size and the large number of proteins analyzed.

**FIGURE 4:**
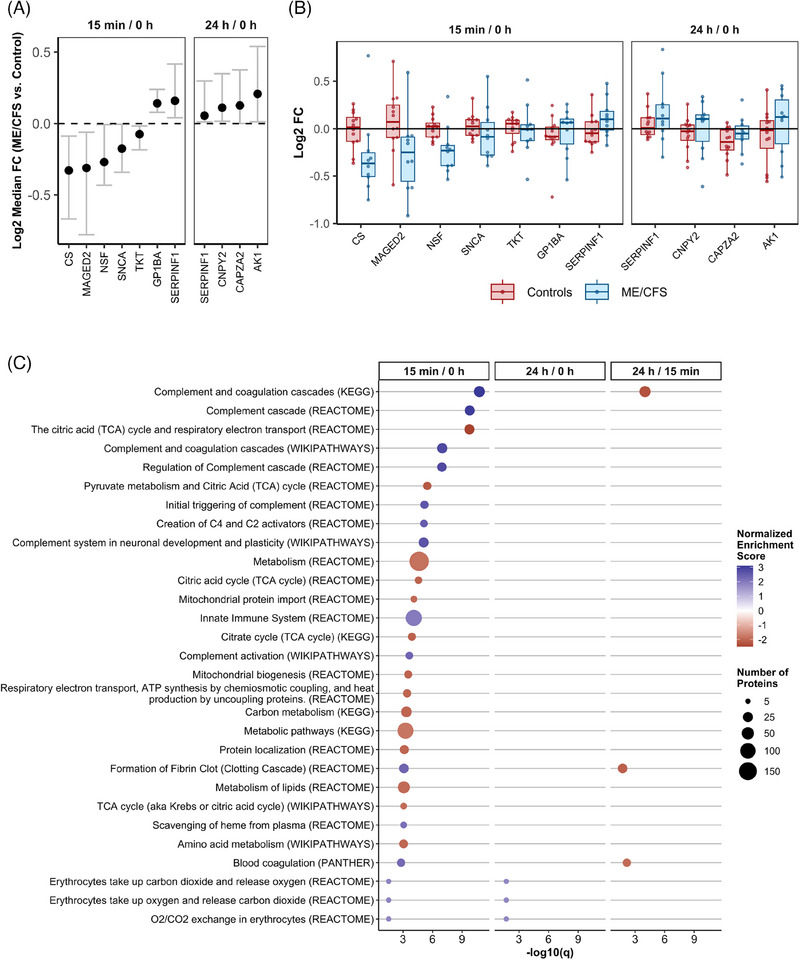
Changes in EV protein levels between ME/CFS and controls over time postexercise. (A) The y‐axis shows the median Log2 fold change (ME/CFS vs. Controls) of 10,000 bootstrapped datasets at each time point for all significant DAPs only. Grey bars with caps show the FDR‐adjusted confidence intervals (*q* < .1). (B) Boxplots of the within‐subject Log2 fold changes for all DAPs in (A). (C) Top 25 pathways significantly enriched in ME/CFS versus controls by gene set enrichment analysis (*q* < .05). Pathways are listed in order of significance at 15 min/0 h postexercise. Bubble size shows the number of compounds in that gene set, and the colour shows the normalized enrichment score (NES).

Consistent with the findings at individual time points, we observed the most differences between ME/CFS subjects and controls in the change from 0 h to 15 min postexercise, which reflects the rapid response phase (Figure 4). Seven proteins exhibited significantly different 15 min/0 h ratios between ME/CFS patients and controls, with five being downregulated in ME/CFS (CS, MAGED2, NSF, SNCA, and TKT) and two being upregulated (GP1BA and SERPINF1) (Figure 4A, *q* < .1). Additionally, four proteins had significantly different 24 h/0 h ratios, all of which were upregulated in ME/CFS. Among these, SERPINF1 (Serpin family F member 1), was found to be significantly upregulated for both the 15 min/0 and 24 h/0 h ratios, although the effect size was smaller for the latter. SERPINF1 is a neurotrophic protein known for its potent inhibition of angiogenesis, which might reflect a compensatory response to the physiological stress induced by exercise.

The within‐subject fold changes used in the bootstrapping analysis are shown in Figure 4B for all significant DAPs, providing insight into the temporal changes over time within each group and subject. The protein with the largest |Log2 median FC| was CS (citrate synthase), which, on average was not altered by exercise in the controls but showed a reduction from 0 h to 15 min postexercise in most ME/CFS patients (Figure 4B). As the first enzyme in the TCA cycle, CS plays a critical role in cellular energy production, and its downregulation may point to impaired energy metabolism in ME/CFS patients.

For SERPINF1, EV levels increased in most ME/CFS patients during the rapid response to exercise and remained elevated 24 h postexercise, in contrast to the minimal changes observed in the controls. This sustained elevation, indicating dysregulation in the rapid response to exercise as well as impaired recovery, may be contributing to PEM experienced by ME/CFS patients.

Capping actin protein of muscle z‐line alpha subunit 2 (CAPZA2) exhibited a different temporal pattern, with reduced levels at 24 h postexercise in most subjects from both groups, although the decrease was more pronounced in the control group compared with patients. CAPZA2 is crucial for regulating the dynamic response of actin filaments, which is essential for muscle contraction, and its dysregulation may reflect impaired muscle recovery or remodelling in the context of ME/CFS.

The box plots in Figure 4B highlight the variability in protein response among individual subjects. ME/CFS patients generally showed greater variability in the fold changes of several proteins compared with controls, such as MAGED2, SNCA, GP1BA, and CNPY2. This increased variability suggests a heterogeneous response to exercise among ME/CFS patients which may reflect differences in disease severity, underlying pathophysiological mechanisms, or individual adaptive capacity.

We performed a quantitative GSEA using the ranked list of proteins derived from the Log2 median FC of the change over time in ME/CFS versus controls. When comparing the 15 min/0 h ratios between groups, we identified 96 significantly dysregulated protein sets (Figure 4C highlights the top 25, *q* < .05; complete results can be found in Table S6). The most significantly altered pathways that differed between groups during the rapid response to exercise included a greater number of complement and coagulation cascade‐related pathways compared with the statistical comparison of proteomic cargo between groups at 15 min postexercise. All complement‐related pathways among the top 25 showed strong positive enrichment, with NES values ranging from 2.3 to 3.1. This indicates that the overactivation of these immune pathways is a hallmark of the rapid response to exercise in ME/CFS, further implicating inappropriate or excessive immune signalling as a key feature of the disease.

The TCA cycle and respiratory electron transport pathway were highly downregulated in the rapid response to exercise in ME/CFS patients compared with controls, with an NES of −2.5, making it the third most significantly altered pathway (Figure 4C). This downregulation of a central metabolic pathway underscores the energy production deficits in ME/CFS patients, which could be a major factor in their inability to sustain physical activity without experiencing severe fatigue and other symptoms. Notably, 11 of the top 25 pathways are related to metabolism, all of which displayed a downregulated rapid response to exercise in ME/CFS versus controls. This widespread metabolic dysregulation in EVs highlights a fundamental impairment in the ability of ME/CFS patients to mobilize energy resources effectively in response to physical stress.

Conversely, the innate immune system pathway, which includes 112 proteins in our dataset, was significantly upregulated in ME/CFS patients compared with controls during the rapid response to exercise, further indicating inappropriate EV immune signalling in these patients. The exaggerated or misdirected activation of the innate immune system in response to exercise may contribute to the chronic inflammation and immune dysfunction observed in ME/CFS, exacerbating symptoms and impeding recovery.

### 3.5 Correlation of EV protein dynamics with disease severity and functional impairment in ME/CFS patients postexercise

**FIGURE 5:**
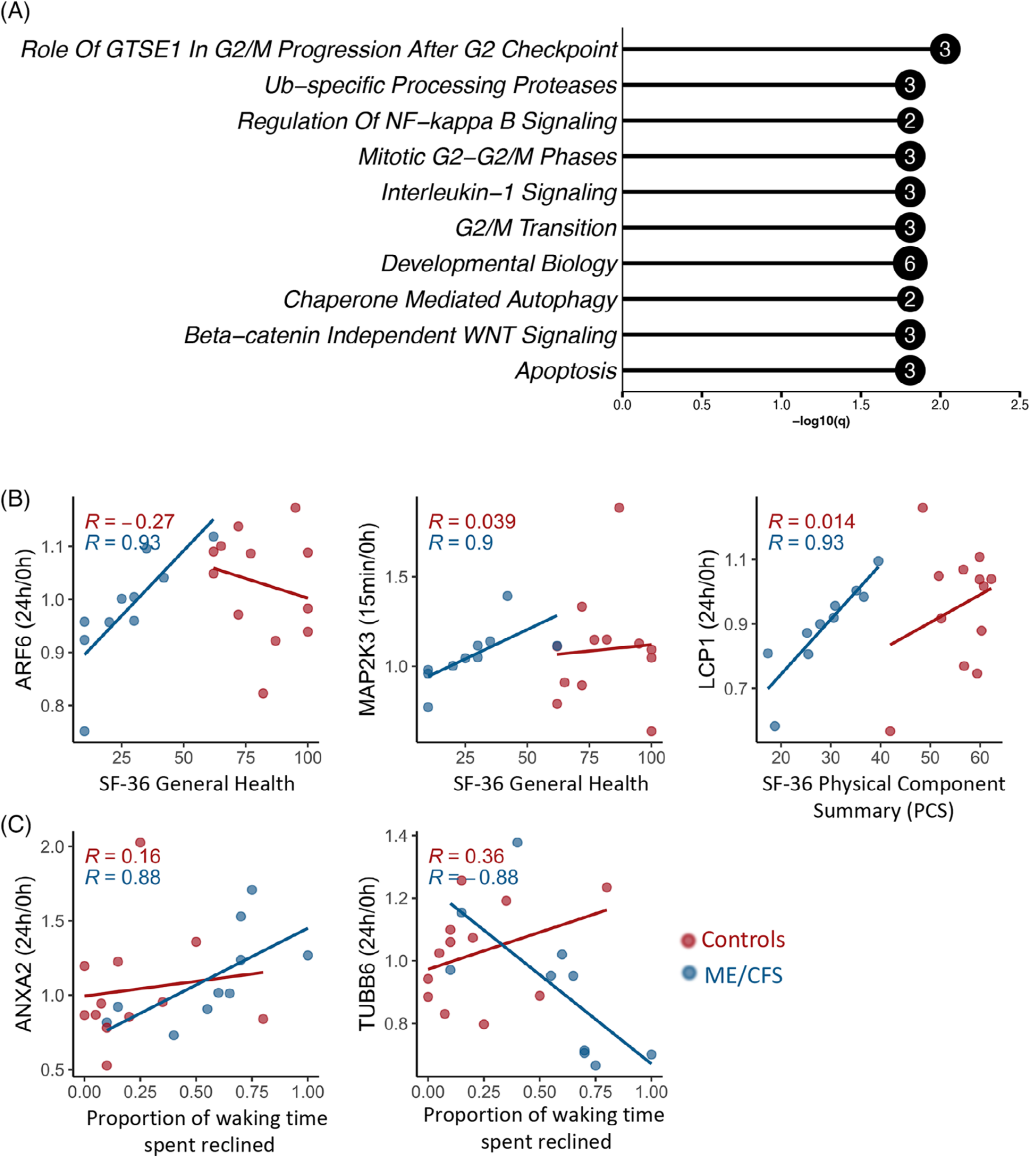
Associations between metrics of overall disease severity and changes in the postexercise EV proteome. (A) Bar plot showing the ‐log10(q) values of the top 10 significant Reactome pathways (*q* < .05) enriched for 18 postexercise EV protein changes that significantly correlate with bell activity scale scores (|R| > .8 and *q* < .1). (B) Correlations between EV protein within‐subject ratios over time and SF‐36 metrics or (C) with the proportion of waking time spent reclined. Each dot is one subject. The lines are linear regression lines for each group, ME/CFS or control. Spearman's R is shown on each plot for controls (red) and ME/CFS patients (blue). All correlations presented are significant in ME/CFS patients (*q* < .1).

In this analysis, Spearman's correlation coefficients were calculated to explore the relationship between temporal dynamics of the 865 EV proteins and metrics of disease severity and disability level, including Bell Activity Scale Scores, SF‐36 scores and the proportion of waking time spent reclined (Figure 5). All correlations were calculated separately for the ME/CFS and control groups.

To ensure the statistical robustness of these correlations, bootstrapped 95% confidence intervals were derived from 2000 resampled datasets using the *bootcorci* R package. *p*‐values were computed to test the null hypothesis that the correlation coefficient is zero, and *q*‐values were obtained using the BH‐FDR correction procedure to account for multiple comparisons. Significant and strong correlations were defined as |*R*| > .8 and *q* < .1. Table S7 shows the correlation coefficients, confidence intervals, and *p‐* and *q‐*values for the correlations that met these criteria.

Changes in the levels of 18 EV proteins from 0 to 24 h in ME/CFS patients showed significant correlations with Bell Activity Scale scores, with 17 proteins positively correlated and one negatively correlated. The significant negative correlation between Desmoglein 1 (DSG1) levels and Bell Scale scores indicates that higher levels of DSG1 in EVs postexercise are associated with more severe disability. DSG1 is a protein primarily involved in cell‐cell adhesion within desmosomes, which are structures that help resist shearing forces and are abundant in cells subjected to mechanical stress.

We investigated pathway enrichment for the set of 18 EV proteins associated with Bell Activity Scale scores using Enrichr. The bar plot presented in Figure 5A highlights the top 10 most significantly enriched Reactome pathways (out of 268 total significant pathways, *q* < .05, Table S8). The enrichment analysis suggests associations between functional disability levels in ME/CFS patients and EV signalling in critical biological processes, including cell cycle regulation, signalling pathways, and apoptosis. Notably, significant pathways such as “Role of GTSE1 in G2/M progression after G2 checkpoint,” “Ub‐specific processing proteases,” and “Regulation of NF‐kappa B signalling” each involve 2 to 3 EV proteins (Figure 5A).

We also found one protein, endoplasmic reticulum protein 29 (ERP29) whose 15 min/0 h ratio had a strong positive correlation with bell activity scale (BAS) scores, such that patients with more severe disability had decreased levels postexercise and more functional patients exhibited an increase postexercise (*R* = .93, Figure S4). ERP29 is a molecular chaperone that participates in protein folding and the ER stress response. This shows the potential failure of the ER stress response in more severe patients.

The 24 h/0 h ratio of ARF6, a GTPase involved in membrane trafficking and cytoskeletal dynamics, and the 15 min/0 h ratio of MAP2K3, a kinase in the MAPK signalling pathway that mediates stress responses, both positively correlated with better general health in ME/CFS patients, as measured by SF‐36 General Health scores. Notably, lymphocyte cytosolic protein 1 (LCP1), which is involved in actin bundling and activation of T cells, showed a positive association between its 24 h/0 h ratio and the SF‐36 Physical Component Summary (Figure 5B; Table S7). ME/CFS patients with an increase in LCP1 in EVs from baseline to 24 h had the highest physical component summary scores, indicating better health.

In contrast, higher 24 h/0 h ratios of Annexin A2 (ANXA2), which is involved in membrane repair, were linked to greater disease severity, as indicated by more time spent reclined. Conversely, a lower 24 h/0 h ratio of TUBB6, a component of microtubules essential for cell structure, was associated with increased time spent reclined (Figure 5C, Table S7).

### 3.6 Postexercise EV proteome alterations correlate with symptom exacerbation in ME/CFS patients

In addition to analyzing overall disease severity, we also examined correlations with specific symptom severity (SSS) scores. Participants rated their symptoms on a scale of 0 to 10 (with 0 indicating no symptoms and 10 indicating very severe symptoms) on the morning of the CPET (0 h) and 24 h after the CPET. We also calculated the change in symptom severity postexercise as the difference between the 0 and 24 h SSS scores (ΔSSS). A positive ΔSSS indicates a worsening of the symptom 24 h postexercise, while a negative ΔSSS indicates an improvement in the symptom over the same period.

**FIGURE 6:**
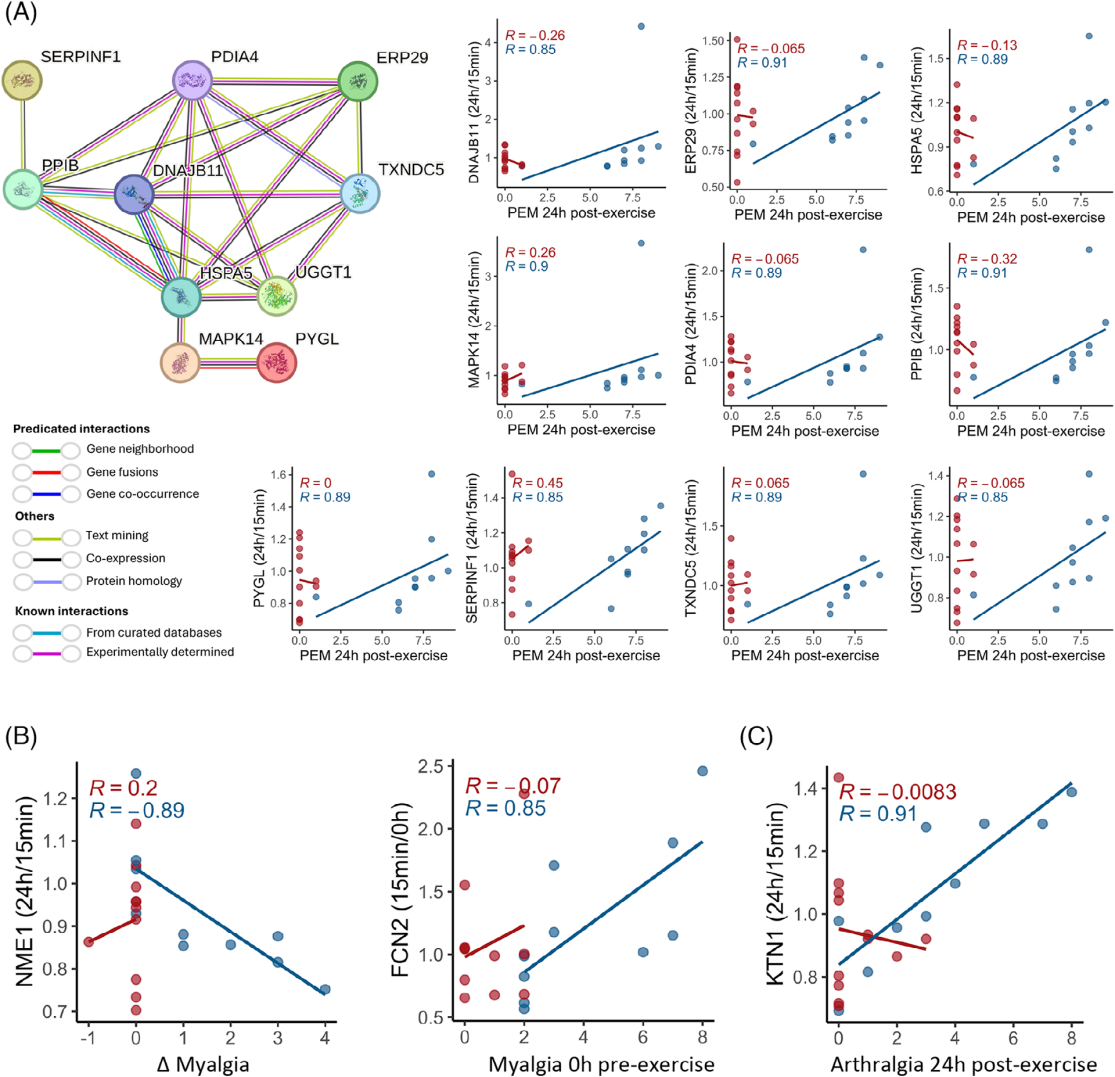
Associations between specific symptom severity and changes in the postexercise EV proteome. (A) PPI network of the 10 proteins correlating with the PEM experienced by subjects 24 h postexercise and corresponding scatter plots. Each node represents a protein, and edges indicate predicted or known interactions. The colour of the edges corresponds to different types of evidence: green lines represent neighbourhood evidence, purple lines represent experimental evidence, blue lines represent co‐occurrence, black lines indicate co‐expression and light green lines denote text mining evidence. The PPI enrichment p‐value is < 1e‐16. (B) Correlations between the change in myalgia from baseline to 24 h postexercise (ΔMyalgia) or Myalgia the day of exercise and within‐subject EV protein ratios over time. (C) Correlations between within‐subject EV protein 24 h/15 min ratios and arthralgia 24 h postexercise. Each dot is one subject. The lines are linear regression lines for each group, ME/CFS or control. Spearman's R is shown on each plot for controls (red) and ME/CFS patients (blue). All correlations presented are significant in ME/CFS patients (*q* < .1).

A core symptom of ME/CFS is PEM, which we found to correlate significantly with changes in several proteins following exercise. Specifically, the 24 h/15 min ratio of 10 proteins showed a positive correlation with the severity of PEM experienced by subjects 24 h postexercise (Figure 6A; Table S9). To further explore relationships between these proteins, we conducted a protein–protein interaction (PPI) network analysis using the STRING platform. STRING (search tool for the retrieval of interacting genes/proteins)^54^ is a bioinformatics tool that predicts and visualizes functional protein associations based on a combination of direct (physical) and indirect (functional) evidence from multiple sources, including experimental data, computational predictions, and literature.

The analysis revealed that these proteins (nodes) form a highly interconnected network, with STRING assigning edge colours to represent different types of interaction evidence, such as known interactions from curated databases, experimentally determined interactions, and predicted interactions based on gene neighbourhood, gene fusions, and co‐occurrence. The PPI enrichment *p*‐value, calculated by comparing the observed network to a random network of the same size drawn from the genome, was less than 1.0 × 10^−16^. This extremely low p‐value indicates that the observed network has significantly more interactions than would be expected by chance, underscoring the potential biological relevance of these proteins in the context of PEM and ME/CFS.

The network diagram illustrates interactions between several key proteins, with DNAJB11, HSPA5, PPIB, and PDIA4 standing out as central hubs (with six or more interactions each). HSPA5, which is involved in protein folding and endoplasmic reticulum (ER) stress responses, and PPIB, a chaperone associated with protein folding, show strong interconnections with other proteins such as PDIA4, MAPK14, and SERPINF1 (Figure 6A). The interaction between SERPINF1 and PPIB suggests a potential role for PPIB in the proper folding and stability of SERPINF1. PDIA4, a member of the PDI family, plays a crucial role in the formation and rearrangement of disulfide bonds within the ER.

We also found that the 24 h/15 min ratio for NME1, a protein involved in nucleoside triphosphate synthesis and cellular stress responses, had a strong and significant negative correlation with the change in myalgia after exercise in ME/CFS patients (Figure 6B). Specifically, when NME1 levels decreased from 15 min to 24 h postexercise (Log2FC < 0), myalgia became worse (Δ > 0), suggesting that NME1‐loaded EVs are involved in signalling pathways that regulate cellular and muscle recovery after stress or injury, including exercise. The change in FCN2 levels from 0 h to 15 min postexercise positively correlated with baseline myalgia (Figure 6B). FCN2 is crucial for innate immune response and complement activation. Patients with increased FCN2 in the rapid response phase had higher muscle pain, which reveals a link between the immune overactivation in ME/CFS patients and the pain they are experiencing. Moreover, the 24 h/15 min ratio for KTN1, a protein involved in intracellular transport and cellular organization, showed a strong and significant positive correlation with arthralgia (joint pain) 24 h postexercise (Figure 6C), which suggests that disruptions in cellular transport processes may be linked to joint pain in ME/CFS patients. All of these correlations were absent in the control group.

### 3.7 Metabolic and protein homeostasis dysregulation linked to unrefreshing sleep postexercise in ME/CFS

The analysis of 24 h/15 min protein level ratios revealed strong and significant positive correlations between 68 proteins and changes in the severity of unrefreshing sleep the night after the CPET in ME/CFS patients (Table S10). Thus, an increase in protein levels from 15 min to 24 h postexercise was associated with an exacerbation of unrefreshing sleep, while a decrease in protein levels corresponded to reduced severity of this symptom.

**FIGURE 7:**
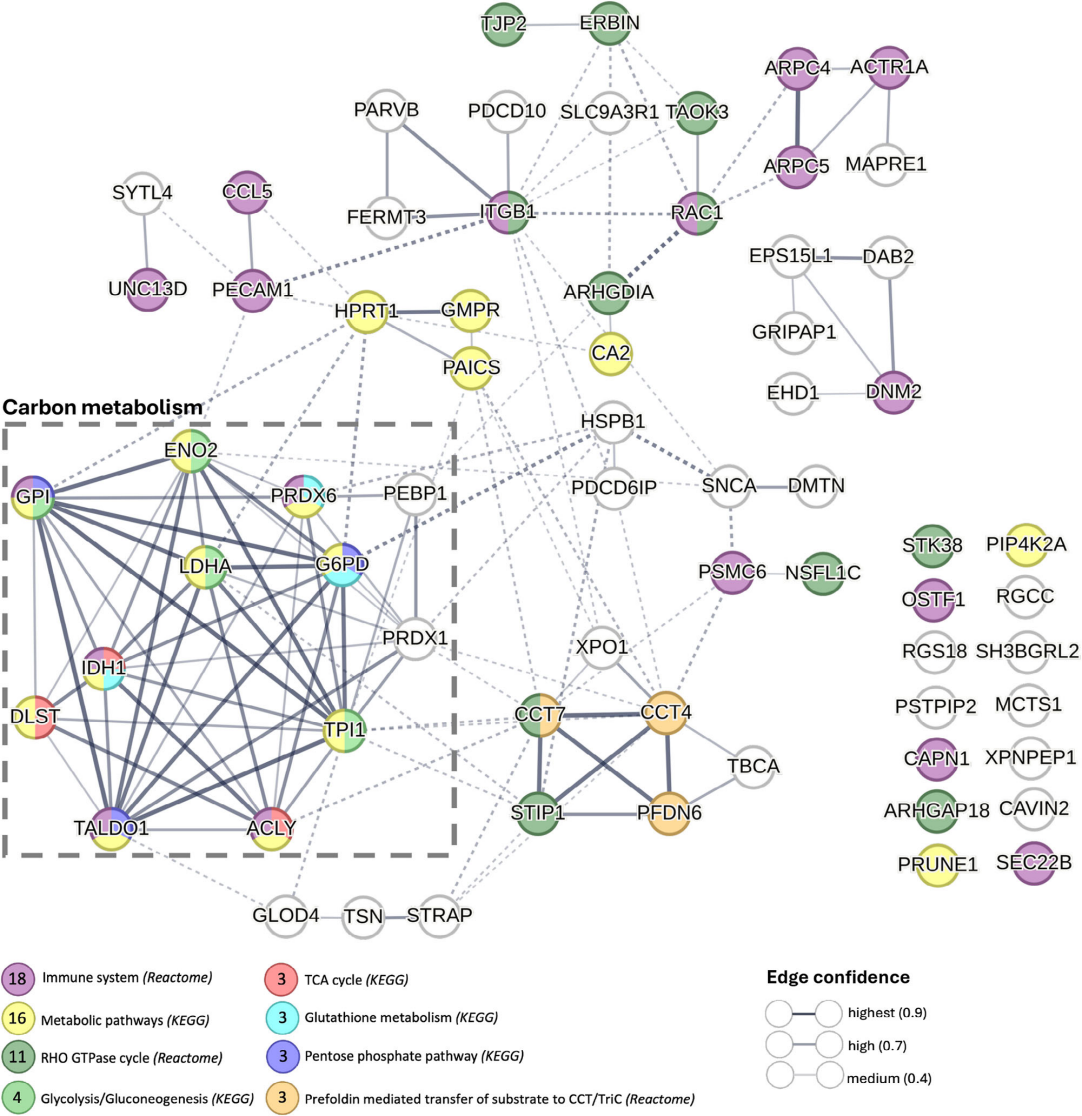
Protein–protein interaction network of EV proteins changes correlating with changes in unrefreshing sleep postexercise. The network was generated using STRING (PPI enrichment *p*‐value < 1e‐16). Each node represents a protein, and the thickness of the edges reflects the confidence level of the predicted interactions, ranging from .4 to .9, with thicker lines indicating higher confidence. Dashed edges indicate speculative or predicted associations that are not as strongly supported by experimental data. The dashed box highlights a cluster of highly interconnected proteins all involved in carbon metabolism. The different colours of the nodes correspond to distinct functional categories or pathways enriched from the KEGG and Reactome databases. The number of proteins belonging to each pathway is shown in the legend.

To further investigate these relationships, we conducted a STRING analysis of PPI involving these 68 proteins (Figure 7, PPI enrichment *p*‐value < 1e‐16). Only 14 of the 68 proteins were not interconnected. The high significance level of connectivity within the network underscores the potential biological relevance of these proteins in the context of unrefreshing sleep and ME/CFS. In the PPI network, the thickness of the edges represents the confidence level of the functional interactions, with thicker edges indicating higher confidence. Dashed edges indicate speculative or predicted associations that are not as strongly supported by experimental data, providing a visual representation of the varying levels of evidence supporting the interactions (Figure 7).

Enrichment analysis using the KEGG and Reactome databases within the STRING platform revealed that 18 of the 68 proteins were associated with the “Immune system,” 16 with “Metabolic pathways,” and 11 with the “RHO GTPase cycle.” A particularly prominent cluster of 12 proteins, enclosed within a dashed box, exhibited high interconnectivity, with edge thickness indicating high to highest confidence (.7–.9) in associations related to carbon metabolism. This cluster includes key metabolic enzymes such as glucose‐6‐phosphate isomerase (GPI), isocitrate dehydrogenase (NADP) cytoplasmic (IDH1), lactate dehydrogenase A (LDHA), and transaldolase 1 (TALDO1). These enzymes are critical to metabolic processes such as “glycolysis/gluconeogenesis,” the “TCA cycle,” “glutathione metabolism,” and the “pentose phosphate pathway” (Figure 7; Table S11).

Notably, three of these proteins, dihydrolipoamide S‐succinyltransferase (DLST), ATP citrate lyase (ACLY), and isocitrate dehydrogenase 1 (IDH1), are directly involved in the TCA cycle. The change in EV levels of all of these proteins during exercise recovery was strongly and positively correlated with less refreshing sleep following exercise. DLST plays a pivotal role in the TCA cycle by facilitating the conversion of succinyl‐CoA to lipoamide. ACLY links the metabolism of carbohydrates and fats by converting citrate into acetyl‐CoA, which is a substrate for both the TCA cycle and fatty acid synthesis. IDH1 catalyzes the conversion of isocitrate to alpha‐ketoglutarate, producing NADPH, which is vital for cellular redox balance. The correlation of these TCA cycle‐related proteins with unrefreshing sleep shows the potential impact of disrupted metabolic processes on sleep quality in response to exercise for ME/CFS patients.

Another significant cluster comprising chaperonin‐containing TCP‐1 subunit 7 (CCT7), chaperonin‐containing TCP‐1 subunit 4 (CCT4), stress‐induced‐phosphoprotein 1 (STIP1), and prefoldin subunit 6 (PFDN6) was linked to the “RHO GTPase cycle” and “prefoldin‐mediated transfer of substrate to CCT/TriC” pathways. This cluster emphasizes the importance of protein folding and cellular signalling in ME/CFS. These proteins play critical roles in maintaining protein homeostasis and proper cellular function, and their dysregulation may contribute to the complex symptomatology of ME/CFS, including unrefreshing sleep.

### 3.8 Exercise performance metrics correlate with EV protein dynamics in controls but not in ME/CFS patients

**FIGURE 8:**
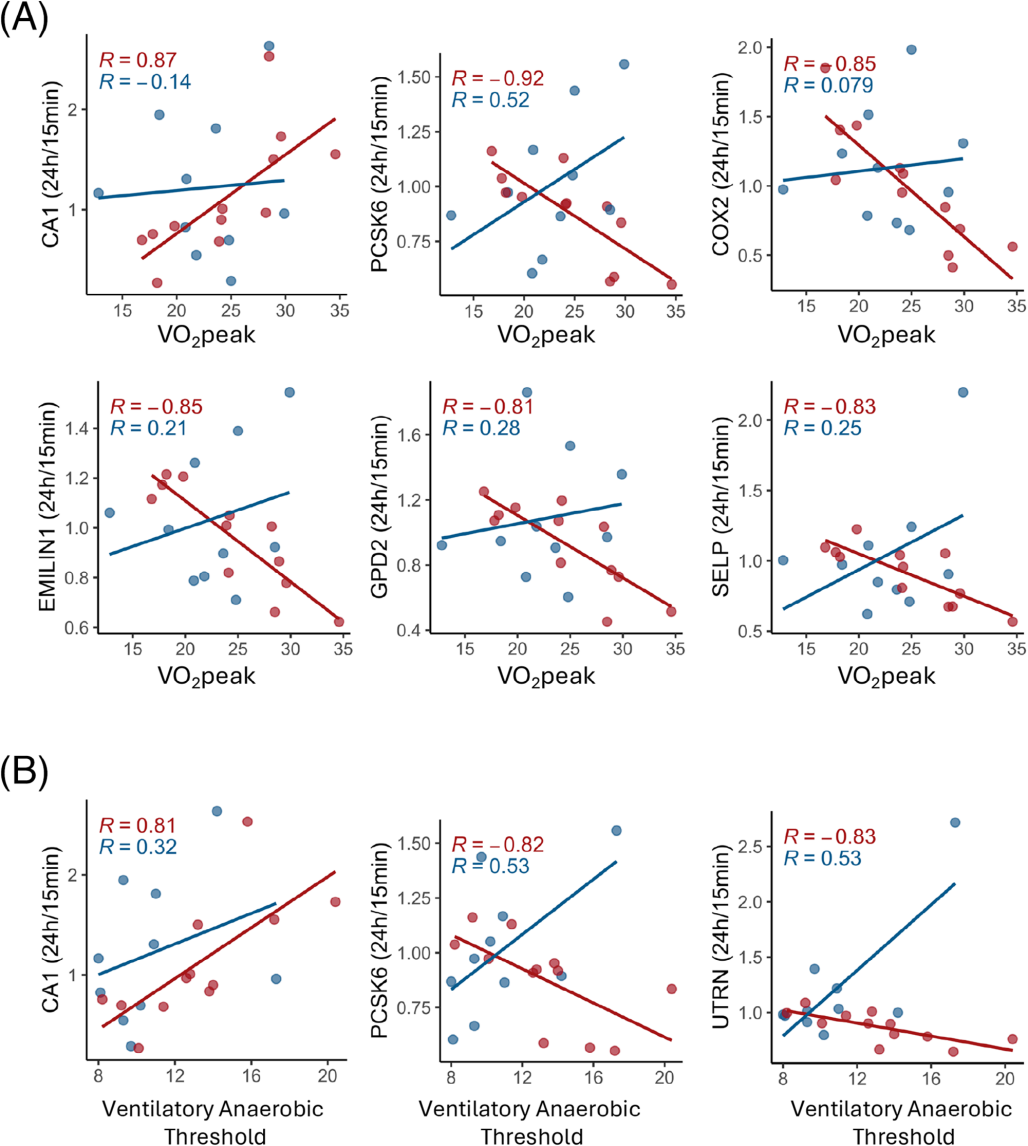
Associations between CPET parameters and changes in the postexercise EV proteome in healthy controls. (A) Associations with VO_2_peak, and (B) with ventilatory anaerobic threshold (VAT). Each dot is one subject. The lines are linear regression lines and Spearman's R are shown on each plot for controls (red) and ME/CFS patients (blue). All correlations presented are only significant in controls (*q* < .1).

We analyzed the correlations between CPET parameters, such as VO_2_peak and VAT, and found significant correlations with postexercise protein levels (24 h/15 min ratios) exclusively in the control group, suggesting a disrupted physiological response in ME/CFS patients (Figure 8). For VO_2_peak, 14 proteins exhibited significant correlations, with 11 showing negative correlations and 3 showing positive correlations (Table S12). Among these, carbonic anhydrase 1 (CA1) stood out with a positive correlation. CA1 is essential for converting CO_2_ to bicarbonate and protons, a process crucial for maintaining acid‐base balance, especially during intense exercise when lactic acid levels rise (Figure 8A). COX2, an enzyme primarily associated with inflammation and oxidative stress, had elevated 24 h/15 min ratios in less fit subjects with lower VO_2_peak and reduced ratios in more fit subjects with higher VO_2_peak. Subjects with better cardiovascular fitness may have adapted to reduce this inflammation during exercise recovery.

PCSK6, EMILIN1, GPD2, and SELP also exhibited negative correlations between their 24 h/15 min ratios and VO_2_peak, with more fit healthy sedentary controls showing decreased levels during the recovery phase. EMILIN1, associated with maintaining vascular integrity, may affect blood flow and oxygen delivery during exercise recovery. GPD2 is involved in glycerol metabolism and energy production and plays a role in mitochondria. SELP is a marker of endothelial activation and inflammation. The absence of these correlations in ME/CFS patients indicates a disrupted physiological response to exercise.

Similarly, for VAT, 6 proteins were significantly correlated, four negatively and two positively (Table S12). CA1 and PCSK6, showed significant correlations with VAT and VO_2_ peak (Figure 8B), reinforcing their roles in both aerobic and anaerobic exercise responses. CA1 positively correlated with VAT, which suggests that its role in acid‐base buffering and metabolic recovery could influence anaerobic metabolism during and after exercise. PCSK6 was negatively correlated with VAT, potentially reflecting its role in postexercise inflammatory or vascular stress pathways, which may influence metabolic recovery and contribute to earlier shifts to anaerobic metabolism during subsequent exercise. Utrophin (UTRN) was also negatively correlated with VAT (Figure 8B). Larger reductions in UTRN in EVs during the recovery phase in fitter individuals could be a part of the healthy muscle repair process, since UTRN is involved in maintaining muscle integrity, particularly in response to stress. These findings suggest that in healthy individuals, these EV proteins play a crucial role in optimizing exercise performance and recovery, while in ME/CFS patients, the dysregulation of these proteins may play a role in their impaired exercise tolerance and prolonged recovery.

## 4 DISCUSSION

### 4.1 Extracellular vesicle concentrations in ME/CFS and following exercise

In this study, we observed a trend towards higher concentrations of circulating EVs in 10 male ME/CFS patients compared with 12 male sedentary controls at baseline and 15 min post‐CPET, though these differences were not statistically significant. This trend is consistent with previous research suggesting elevated EV levels in ME/CFS, potentially indicating an underlying chronic inflammatory state.^26^, ^27^, ^28^, ^29^, ^30^, ^31^ Similar increases in EV numbers have been documented in other chronic conditions, such as liver disease,^60^ Parkinson's disease,^61^ and cardiovascular diseases.^62^, ^63^

The literature on exercise‐induced changes in EV concentrations in healthy males is varied, with some studies reporting significant increases following exercise, while others did not observe such changes. For example, Whitham et al.^21^ found a significant increase in the concentration of circulating EVs following a 1 h cycling session, and Annibalini et al.^64^ reported increased EV concentrations 2 h after resistance training. Similarly, Fruhbeis et al.^20^ observed a significant increase in EV numbers immediately postexercise during an incremental cycling test, with levels returning to baseline within 90 min. In contrast, Lovett et al.^65^ found no significant changes in EV size or number following muscle‐damaging exercise.

In our sedentary male control cohort, we observed a significant increase in EV concentration 24 h postexercise, but not at 15 min (Figure 2A). This exercise‐induced increase was not observed in the ME/CFS patients. This is consistent with our finding of decreased abundance of proteins postexercise involved in vesicle‐mediated transport and biogenesis in ME/CFS (Figure 3B), which suggests an impaired ability to produce EVs in response to exercise. Our group recently published a paper analyzing EV proteomics in females during exercise, and we observed the same pattern in female sedentary controls and female ME/CFS patients, but with a significant increase in controls at 15 min postexercise and a further increase at 24 h.^28^ The smaller sample size in the current study is likely why the increase in control males at 15 min did not reach significance. The diversity of temporal concentration profiles across studies highlights the complexity of EV dynamics in response to exercise and suggests that variations in subject characteristics, health status, the type of exercise, and the timing of EV measurements play crucial roles in these outcomes.

### 4.2 Overactivation of the complement system in ME/CFS

Our analyses revealed significant pathway alterations in male ME/CFS patients compared with controls, particularly 15 min postexercise. Among the most significant pathways, the complement and coagulation cascades consistently stood out across three different databases, with EV proteins in these pathways upregulated in patients compared with controls. While differences in the proteins in the leading‐edge subset (e.g., C1q, C3, C6) were not significant on the individual protein level, as a group they were overactivated in ME/CFS versus controls. C1q is the first component of the classical complement pathway and plays a crucial role in innate immunity. C3, a central member of the cascade, has beneficial roles in promoting phagocytosis of pathogens, but its overactivation leads to host cell damage.^66^ C6 is an early component of the membrane attack complex assembly, responsible for lysing targeted cells and contributing to inflammasome activation during infection.^67^

Notably, the complement cascade was the only pathway significantly enriched both at baseline and 15 min postexercise (Figure 3C). This finding suggests that the complement system in ME/CFS patients may be primed for overactivation even before exercise, and this heightened immune response may contribute to the PEM that patients experience. The overactivation was further evidenced by a significant increase in complement pathways for the 15 min/0 h within‐subject fold changes in patients versus controls, demonstrating an intensified complement response during the rapid postexercise phase in ME/CFS (Figure 4C).

We also observed a positive correlation between the change in EV ficolin‐2 (FCN2) levels from 0 h to 15 min postexercise and the severity of myalgia at 0 h (Figure 6B). FCN2 belongs to the lectin pathway of complement activation. This increase may propagate inflammatory signals and potentially contribute to the persistence of chronic muscle pain in ME/CFS. Interestingly, EVs containing FCN2 have been implicated in osteoarthritic pain as well^68^ and FCN2 is increased in plasma‐derived EVs following percutaneous coronary intervention.^69^ Additionally, FCN2 has been identified as an early predictor of myocardial infarction^70^ further underscoring its broader role in inflammatory and pain‐related conditions, including ME/CFS.

The detection of these key complement factors within EVs aligns with previous findings that EVs can carry complement components and potentially modulate immune responses.^71^, ^72^ In ME/CFS, the presence of these complement‐containing EVs might suggest a mechanism through which immune dysregulation is perpetuated, particularly following physical exertion. The activation of the complement system by EVs, by C1q binding directly to lipid membranes, could lead to increased inflammation and cellular damage, which may contribute to PEM. Elevated levels of complement components such as C1q and C3 have also been found in EVs from other inflammatory conditions, like multiple sclerosis and ischemic stroke,^73^, ^74^ which may indicate a shared pathway of immune‐mediated damage.

In contrast, in our study of female sedentary controls and ME/CFS patients, we found the complement system to be inhibited 15 min postexercise.^28^ This discrepancy suggests sex differences in complement system activation in ME/CFS, which could have implications for developing sex‐specific treatments. The inhibition observed in females may indicate a different immune response mechanism, perhaps related to hormonal influences or other sex‐specific factors that warrant further investigation.

While there are no other studies reporting changes in complement proteins in EVs in ME/CFS patients, there are two studies that examined baseline plasma levels of complement cascade proteins.^75^, ^76^ Castro‐Marrero et al.^75^ reported that C3 baseline plasma levels were elevated in severe and moderate ME/CFS female cases compared with mild ones, and a significant subgroup (42.8%, 107 out of 250 female patients) exhibited increased circulating levels of C1q. There has been no similar study published looking at male ME/CFS patients. However, Nunes et al.^76^ found no significant difference in baseline plasma C1q levels between groups, instead noting downregulation of C1s and upregulation of C6 in the ME/CFS group (15 ME/CFS patients versus 10 controls, 72% female).

This overactivation of the complement system, whether in EVs or plasma, might also contribute to the chronic nature of the disease, as the immune system remains in a heightened state of alert, potentially causing ongoing tissue damage and inflammation. EVs may serve as a vehicle for disseminating proinflammatory complement signals throughout the body, especially post‐exertion. The variability in complement component levels observed across different studies highlights the need for further research to clarify these mechanisms and how they contribute to the heterogeneous nature of ME/CFS, including sex‐specific aspects of the disease.

### 4.3 Metabolic impairment in ME/CFS patients’ EVs postexercise

In our analyses, we identified numerous differentially abundant proteins (DAPs) between ME/CFS patients and controls, many of which are involved in the tricarboxylic acid (TCA) cycle and other metabolic processes. The TCA cycle and respiratory electron transport pathways were significantly downregulated in ME/CFS patients in response to exercise, compared with controls (Figures 3C and 4C). The change in citrate synthase (CS), the first enzyme in the TCA cycle, from baseline to 15 min postexercise was significantly different in controls and patients (Figure 4A). CS showed the largest reduction from baseline to 15 min postexercise in ME/CFS patients, whereas on average a change postexercise was not seen in controls (Figure 4B). Additionally, changes from 15 min to 24 h postexercise in DLST, ACLY, and IDH1 (proteins also linked to the TCA cycle), were strongly correlated with changes in the degree of unrefreshing sleep following exercise (Figure 7).

A metabolomic analysis of baseline plasma samples from a Japanese cohort, comprising 67 ME/CFS patients and 66 controls, revealed significantly decreased citrate levels in ME/CFS patients comparedwith healthy controls.^77^ Significant reductions in citrate synthase, succinate reductase, and cytochrome‐c oxidase were also observed in anterior tibialis and right quadriceps muscle biopsies from CFS patients.^78^, ^79^ These results support the notion that impaired energy metabolism is a critical factor in ME/CFS, as evidenced by several metabolomic studies showing alterations in key pathways, such as the TCA cycle and amino acid metabolism.^77^, ^80^, ^81^ Our group has previously measured plasma metabolites longitudinally before and after CPET in subjects from the same cohort as the current study (71% females).^13^ Although we found the most differences in energy metabolism pathways in female ME/CFS patients, in male ME/CFS patients we observed a significant decrease in the TCA cycle metabolite alpha‐ketoglutarate 15 min postexercise that was not seen in sedentary controls. Thus, there is evidence of downregulation of the TCA cycle in both plasma and EVs in male ME/CFS patients immediately postexercise.^13^

We also observed strong downregulation of many other metabolism‐related pathways 15 min postexercise, including general metabolism, valine, leucine, and isoleucine degradation, as well as fatty acid and lipid metabolism (Figures 3, 4). These pathways are also dysregulated in female ME/CFS patients’ plasma and urine metabolite profiles 24 h after exercise challenge,^13^, ^14^ and oleoylcholine, involved in fatty acid metabolism, was a key feature in classifying patients versus controls with a random forest model,^82^ but metabolic alterations have been less studied in male patients. Valine, leucine, and isoleucine are branched‐chain amino acids (BCAAs) known to stimulate muscle protein synthesis by activating the mTOR signalling pathway, which is crucial for muscle growth and repair.^83^ BCAAs are abundant in skeletal muscle, where most BCAA catabolism occurs.^84^ There are no studies specifically studying the EV metabolome postexercise challenge, but EVs can mediate the transfer of metabolic information between cells, potentially transporting amino acids or their derivatives to modulate metabolic pathways like protein synthesis or energy production in recipient cells. For example, prostate cancer‐associated fibroblasts have been shown to secrete exosomes containing amino acids like glutamine, arginine, and leucine that regulate the metabolism of recipient cancer cells.^85^

The strong and significant positive correlations between 68 proteins and changes in the degree of unrefreshing sleep following exercise in ME/CFS patients revealed a PPI network with strong interconnections between proteins related to carbon metabolism (Figure 7). Specifically, key metabolic enzymes such as GPI, LDHA, and TALDO1, which are critical to glycolysis, glutathione metabolism, and the pentose phosphate pathway (Figure 7; Table S11), strongly correlated with the degree of unrefreshing sleep. Healthy males have previously shown increased levels of proteins related to glycolysis in EVs immediately postexercise.^21^

Altogether, reduced levels of proteins with important metabolic functions in patients’ EVs following exercise compared with controls may indicate a failure in ME/CFS patients to mount an adequate metabolic signalling response following exercise. Perhaps the dysregulated metabolic responses in ME/CFS patients, which impair energy production and cellular repair mechanisms, are also ultimately contributing to the pathophysiology of unrefreshing sleep after exercise. The widespread metabolic dysregulation observed underscores a fundamental impairment in the ability of ME/CFS patients to effectively mobilize energy resources in response to physical stress. Targeting these disrupted pathways in EVs could offer new therapeutic opportunities.

### 4.4 Endoplasmic reticulum stress and protein misfolding are dysregulated in ME/CFS and associated with PEM

We found 10 proteins whose 24 h/15 min ratios positively correlated with the severity of PEM in ME/CFS patients at 24 h postexercise (Figure 6A). These proteins form a highly interconnected PPI network related to endoplasmic reticulum (ER) stress management and protein folding. Several of these proteins were also found to be differentially abundant between ME/CFS patients and controls. Specifically, the 15 min/0 and 24 h/0 h ratios of SERPINF1 are elevated in patients (Figure 4A,B). Thioredoxin domain‐containing protein 5 (TXNDC5) levels were significantly decreased in patients both at baseline and 15 min postexercise, and PDIA4 levels were also decreased at 15 min (Figure 3A).

Additionally, endoplasmic reticulum protein 29 (ERP29) 15 min/0 h ratios were positively correlated with BAS score, so a larger change from baseline to 15 min postexercise occurred in less disabled patients, whereas more disabled patients showed a decrease in ERP29 postexercise (Figure S4). Finally, we found that four proteins whose 24 h/15 min ratios correlated with the change in unrefreshing sleep postexercise, CCT7, CCT4, STIP1, and PFDN6, also function in protein folding and maintaining homeostasis between protein synthesis and degradation, particularly in the prefoldin‐mediated transfer of substrate to “CCT/TriC” pathway.

The presence of HSPA5 (a master regulator of ER homeostasis), PPIB, DNAJB11 (an ER protein that is a co‐chaperone for HSPA5), and PDIA4 as central hubs in the PPI network (Figure 6A) suggests that disruptions in protein folding and ER stress management may play a significant role in the pathophysiology of PEM. The involvement of PDIA4, TXNDC5, and ERP29, all members of the PDI family, underscores the importance of proper protein folding and cellular stress responses in maintaining homeostasis under conditions of chronic or post‐exertional stress.^86^, ^87^ PDIs are enzymes that facilitate the formation, breakage, and rearrangement of disulfide bonds, ensuring correct protein folding. They have emerged as critical players in health and disease, especially in neurodegenerative conditions such as Alzheimer's disease (AD), Parkinson's disease (PD), and amyotrophic lateral sclerosis (ALS) which are characterized by chronic ER stress, improper protein folding and the accumulation of abnormal protein inclusions.^88^, ^89^ Increased levels of PDIs have been observed in the spinal cord and cerebrospinal fluid of ALS patients.^90^

We found reduced PDIA4 and TXNDC5 levels in EVs at baseline (Figure 3A). When combined with the correlation between the change in their levels during recovery and greater severity of PEM (Figure 6A), this suggests that impaired protein folding prior to exercise is then exacerbated during exercise recovery, contributing to PEM. PDIA4 is involved in inflammation in skeletal muscle,^91^ adipose tissue,^92^ and endothelial cell function.^93^ Additionally, the interaction between SERPINF1 and PPIB (Figure 6A) may have critical implications for both neuroprotection and vascular function in ME/CFS. SERPINF1's known antiangiogenic activity^94^ could be influenced by PPIB‐mediated protein folding, potentially affecting vascular stability and function during PEM.

The ER plays a role in EV biogenesis,^95^ and ER stress may alter EV biogenesis and cargo in vitro.^96^, ^97^ While there is accumulating evidence of ER‐associated proteins in EVs in various normal and pathophysiological states, the mechanisms and role of these proteins in EVs, including in states of cellular stress such as postexercise, have not been well characterized yet.^98^ We also found postexercise dysregulation of ER‐associated proteins and PDI family members in EVs in female ME/CFS patients. Significant DAPs with decreased levels in EVs of female ME/CFS patients compared with controls at 15 min postexercise included HSPA5, PPIB, PDIA6, and HSP90B1. PDIA6 also showed reduced 15 min/0 and 24 h/0 h ratios in patients, while PPIB had increased 24 h/15 min ratios.

To the best of our knowledge, only one study showed a maladaptive ER stress response in ME/CFS, in muscle tissue.^99^ Authors found decreased levels of HSPA5 (also known as BiP/GRP78) along with increased levels of ER stress marker eukaryotic translation initiation factor 2 alpha kinase 3 (EIF2AK3/PERK) in skeletal muscle tissue from ME/CFS patients compared with controls, which is consistent with ER stress response failure. Simultaneously, they found an increase in Wiskott‐Aldrich syndrome protein family member 3 (WASF3), which is regulated by the ER stress response, and showed that transgenic mice overexpressing WASF3 exhibited impaired exercise tolerance. WASF3 was detected in EVs in this study, but it was not detected in enough samples to meet the criteria for analysis.

Altogether, these findings show that dysfunctional ER stress responses and impaired protein folding in EVs pre‐ and postexercise are associated with PEM in ME/CFS patients. The involvement of multiple ER‐associated and PDI‐family proteins underscores the importance of maintaining ER homeostasis and the observation that these disruptions occur in both sexes, in EVs and skeletal muscle, links them to PEM, disability, and unrefreshing sleep. While our results, along with prior evidence, support a potential role for impaired ER stress responses in driving PEM, further studies are needed to determine causality in patients. Collectively, this highlights ER stress and protein folding as key therapeutic targets warranting further investigation.

### 4.5 Disability severity linked to specific EV protein changes postexercise in ME/CFS

In this study, multiple correlations were observed between EV protein levels and metrics of disability or overall disease severity. These correlations were not present in healthy individuals. Changes in the levels of 18 proteins from 0 to 24 h in ME/CFS patients significantly correlated with BAS scores, with only one of those proteins, DSG1 (Desmoglein 1), negatively correlated (Figure 5). This suggests that higher postexercise levels of DSG1 in EVs are associated with more severe disability. Desmoglein 1 is an essential component of desmosomes, structures that mediate cell–cell adhesion and confer tissue strength, especially in mechanically stressed tissues like the heart.^100^ Thus, disruptions in tissue integrity and mechanical resilience might contribute to disability in ME/CFS. The remaining positively correlated proteins were linked to processes such as cell cycle regulation, protein degradation, and immune signalling, including the “Role of GTSE1 in G2/M progression after G2 checkpoint,” “Ub‐specific processing proteases,” “interleukin‐1 Signalling”, and “Regulation of NF‐kappa B signalling” pathways (Figure 5A). These findings are consistent with our earlier study on females, which showed that increased DAPs in ME/CFS patients 15 min postexercise were primarily involved in apoptosis and cell cycle checkpoint pathways, although we did not find the same correlations in that study.^28^

We observed that the 24 h/0 h ratio of ANXA2 in EVs positively correlated with the proportion of waking time spent reclined in individuals with ME/CFS, while the same ratio for TUBB6 exhibited a negative correlation (Figure 5C). The proportion of waking time spent reclined is another metric of overall disease severity, with more severely disabled patients spending more time reclined. ANXA2 is a protein involved in membrane‐related processes, cellular stress responses, and inflammation.^101^, ^102^ The increase in ANXA2 at 24 h versus baseline in more severely disabled patients may suggest that ANXA2 is involved in abnormal recovery processes.

We previously identified ANXA2 in the altered EV proteomic profile of ME/CFS female patients and linked it to broader dysregulation of immune and inflammatory responses.^28^ We found elevated levels of ANXA2 in female ME/CFS patients’ EVs at 15 min postexercise, further supporting the idea that this protein may play a role in modulating the body's maladaptive response to exertion in ME/CFS. Elevated levels of ANXA2 in EVs may reflect the body's heightened effort to manage stress and inflammation during the post‐exertional phase, potentially contributing to the prolonged and exacerbated symptoms observed in PEM.

In contrast, TUBB6, a tubulin protein critical to the cytoskeletal organization, showed a negative correlation with the proportion of waking time spent reclined. The decrease of TUBB6 24 h postexercise in more severely disabled patients could indicate impaired cytoskeletal adaptation during exercise recovery. Although TUBB6 was not detected in the female study, which measured fewer proteins overall, the 24 h/0 h ratios of several other tubulin proteins were negatively correlated with the severity of female ME/CFS patients’ myalgia and arthralgia (muscle and joint pain) postexercise.^28^ Also, we have previously found plasma TUBA1B/A/C levels to be part of a group of proteins able to classify ME/CFS patients versus controls (86% accuracy).^29^ Taken together, these results indicate widespread dysregulation of tubulin proteins in both plasma at baseline and in EVs postexercise.

Overall, the absence of these correlations in healthy controls indicates they are pathological responses specific to ME/CFS. Taken together, we saw increases in levels of several proteins involved in the immune system at 24 h postexercise in ME/CFS patients with higher functional capacity and less severe disability (i.e., “Regulation of NF‐kappa B signalling” and “Interleukin‐1 signalling” with BAS score, and LCP1 with SF‐36 general health score (Figure 5B)). Although one immune system protein, ANXA2, was decreased postexercise in patients with higher functional capacity (less time spent reclined). The immune system's crucial role in a healthy exercise response is compromised in these patients, potentially contributing to their exercise intolerance. More research is needed to understand the role of these EV proteins in exercise recovery, and how their activity may be different in healthier versus more disabled individuals.

### 4.6 Exercise physiology metrics correlate with EV protein ratios postexercise in controls but not in ME/CFS patients

In healthy controls, the correlations between the CPET parameters VO₂peak or VAT, related to cardiovascular fitness, and several 24 h/15 min EV protein ratios suggest that EV‐mediated protein dynamics are integral to postexercise recovery and optimal performance (Figure 8). However, in ME/CFS patients, the absence of such correlations reflects a significant disruption in these physiological processes.

Notably, EMILIN1, a protein crucial for maintaining vascular integrity and regulating resting blood pressure,^103^, ^104^ was found to be negatively correlated with VO₂peak, such that individuals with better cardiovascular fitness decreased EV EMILIN1 levels during exercise recovery. Thus, higher levels of EMILIN1 in EVs may be linked to vascular stiffness or reduced blood flow, potentially impairing oxygen delivery after physical exertion (Figure 8A). In our previous study on female EV proteomics postexercise, we observed that EMILIN1 levels were significantly higher in ME/CFS patients compared with healthy controls 24 h after exercise,^28^ suggesting that in ME/CFS patients, the elevated EMILIN1 may reflect a more prolonged vascular response to exercise‐related stress, which may contribute to patients’ exercise intolerance.

The negative correlation of mitochondrial enzyme GPD2, which is involved in glycerol metabolism, with VO₂peak suggests that a shift towards less efficient energy production pathways may occur when higher levels of GPD2 are present. Elevated GPD2 levels could indicate a greater dependency on glycerol as an energy source, which may be less efficient in supporting prolonged physical exertion, contributing to lower aerobic capacity. This aligns with other studies showing metabolic dysregulation in ME/CFS, including impaired glucose handling and fatty acid oxidation,^7^, ^8^, ^105^, ^106^, ^107^ both of which are crucial for maintaining aerobic exercise capacity.

The observed correlations indicate that in healthy individuals, EVs may act as mediators that enhance physiological resilience during exercise recovery by supporting vascular, metabolic, and inflammatory responses. The absence of significant correlations between CPET parameters and EV protein ratios in ME/CFS patients highlights a key difference in their physiological response to exercise in comparison to healthy individuals. Previous studies have demonstrated that ME/CFS patients experience abnormal metabolic responses during and after exercise, including lower VO₂peak, earlier VAT, and prolonged post‐exertional malaise.^108^ In our study, the lack of correlation between EV protein ratios and CPET parameters suggests that the normal adaptive roles of EVs, such as buffering acid‐base balance, regulating vascular integrity, and modulating metabolic pathways, are disrupted in ME/CFS patients.

## 5 LIMITATIONS

The primary limitation of this study is the small sample size. While comparable to other longitudinal studies of plasma EVs in acute exercise (Darragh, O'Driscoll, and Egan 2021),^21^ it remains limited to a study of ME/CFS, a complex and heterogeneous disease. Due to the inherent variability of the nanoLC‐MS/MS TMT experiments, not all proteins are detected across all experiments and samples. To address missing data, imputation was performed using random forest after filtering out proteins with more than 25% missing values. While this is a standard and robust imputation approach for mass spectrometry proteomics studies, in which missing data is to be expected, it is possible that it could increase the risk of false discoveries. Additionally, a bootstrapping statistics approach was required to compare experimental groups instead of combining data from all TMT experiments. Throughout analyses, we applied stringent thresholds for false discovery rate adjustment of confidence intervals and p values to mitigate these limitations. Another limitation is that only ME/CFS patients capable of completing the exercise protocol were included, excluding those who are severely disabled or bedbound and unable to perform the cycling test. However, we included patients with a range of symptoms and overall disease severity/disability, which enabled us to identify correlations between these clinical data and the changes in EV protein levels post‐exertion. A standardized exertion for both patients and controls was essential to study the molecular response to exertion and PEM in male ME/CFS patients. Additionally, the plasma samples for this study were not platelet‐free, which may have resulted in the ex vivo release of platelet‐derived EVs.^109^, ^110^ Nonetheless, the consistent sampling protocol across all individuals enables us to report specific exercise‐induced effects and compare the EV proteomic profile of ME/CFS patients with those of sedentary controls. Finally, while a similar study was previously conducted in female ME/CFS patients and sedentary controls,^28^ a direct comparison between male and female EV proteomics postexercise is not possible, as these studies were conducted years apart. The current study in males, which was completed more recently, detected a greater number of proteins due to advancements in the analytical platform.

## 6 CONCLUSION

This study revealed profound dysregulation in EV proteomic cargo before and after exercise in male ME/CFS patients compared with sedentary male controls. We also observed disrupted EV protein dynamics in response to exertion. Particularly prominent findings include the downregulation of protein sets including the TCA cycle and other metabolic pathways in ME/CFS patients 15 min postexercise, alongside the upregulation of complement system proteins. Additionally, we found the dynamics during exercise recovery of 10 EV proteins involved in protein folding and the ER stress response were highly correlated with the severity of PEM in ME/CFS patients. Several of these proteins also displayed significantly altered levels compared with controls. Thus, the response to exercise in male ME/CFS patients’ EV proteome is characterized by an inadequate metabolic and ER stress response combined with heightened immune system activation.

## AUTHOR CONTRIBUTIONS

Katherine A. Glass and Ludovic Giloteaux contributed to conceptualization, formal analysis, investigation, methodology, visualization, and writing of the original draft, review and editing, with both taking the lead in these areas. Sheng Zhang provided supporting contributions to methodology, resources, software, and review and editing. Maureen R. Hanson led the conceptualization, secured funding, managed the project, provided resources and supervision, and contributed to reviewing and editing.

## CONFLICT OF INTEREST STATEMENT

The authors declare no conflict of interest.

## CONSENT FOR PUBLICATION

Not applicable

## ETHICS STATEMENT

Informed consent was obtained from all participants and all protocols were approved by the Ithaca College Institutional Review Board (protocol 1017‐12Dx2), and the Weill Cornell Medical College Institutional Review Board (protocol 1708018518). The research adhered to the tenets of the Declaration of Helsinki.

## Supporting information



Supporting Information

Supporting Information

## Data Availability

The protein abundance data for each protein and subject, along with the corresponding phenotype data, are available on mapMECFS, an interactive portal that offers access to research results from ME/CFS‐focused studies at https://www.mapmecfs.org. Additional data from our analyses can be found in the Supporting Information Tables.
