# Supplementary material for: Extracellular vesicle proteomics uncovers energy metabolism, complement system, and endoplasmic reticulum stress response dysregulation postexercise in males with myalgic encephalomyelitis/chronic fatigue syndrome
Source: Clin Transl Med. 2025 Jun 4;15(5):e70346. doi: 10.1002/ctm2.70346 (PMC12135887; doi:10.1002/ctm2.70346)
Supplement: Supplementary file 1 — Supporting Information [file CTM2-15-e70346-s002.pdf]

Supplementary Figure S1

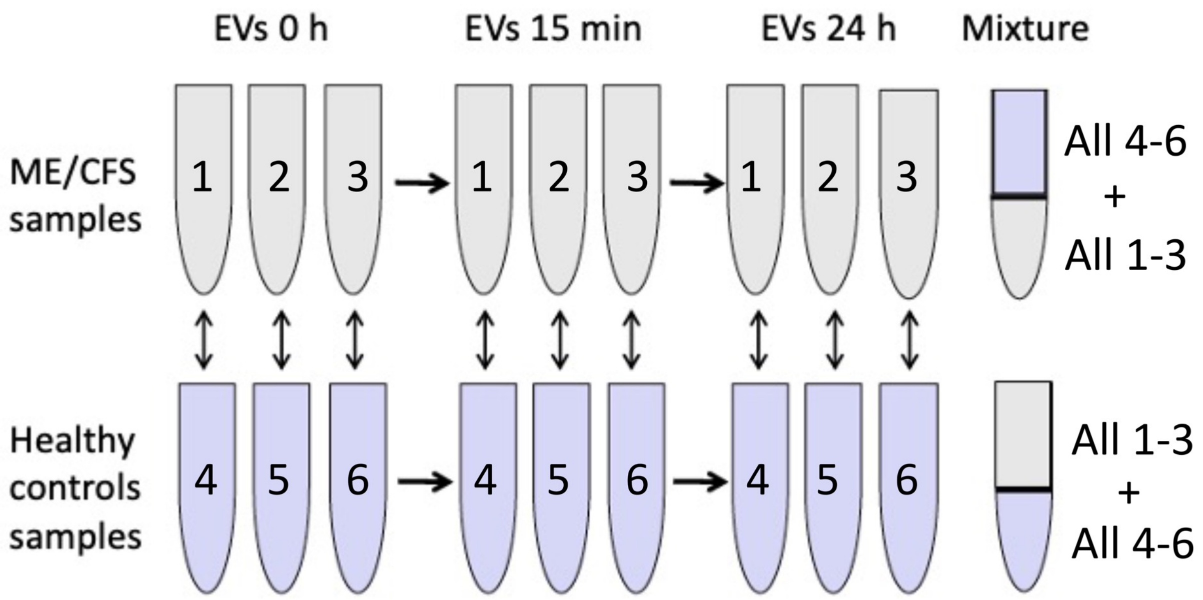

Supplementary Figure S1: TMT10-plex analysis strategy. 1, 2, and 3 represent three different ME/CFS subjects. 4, 5, and 6 represent three different healthy control samples.

Supplementary Figure S2

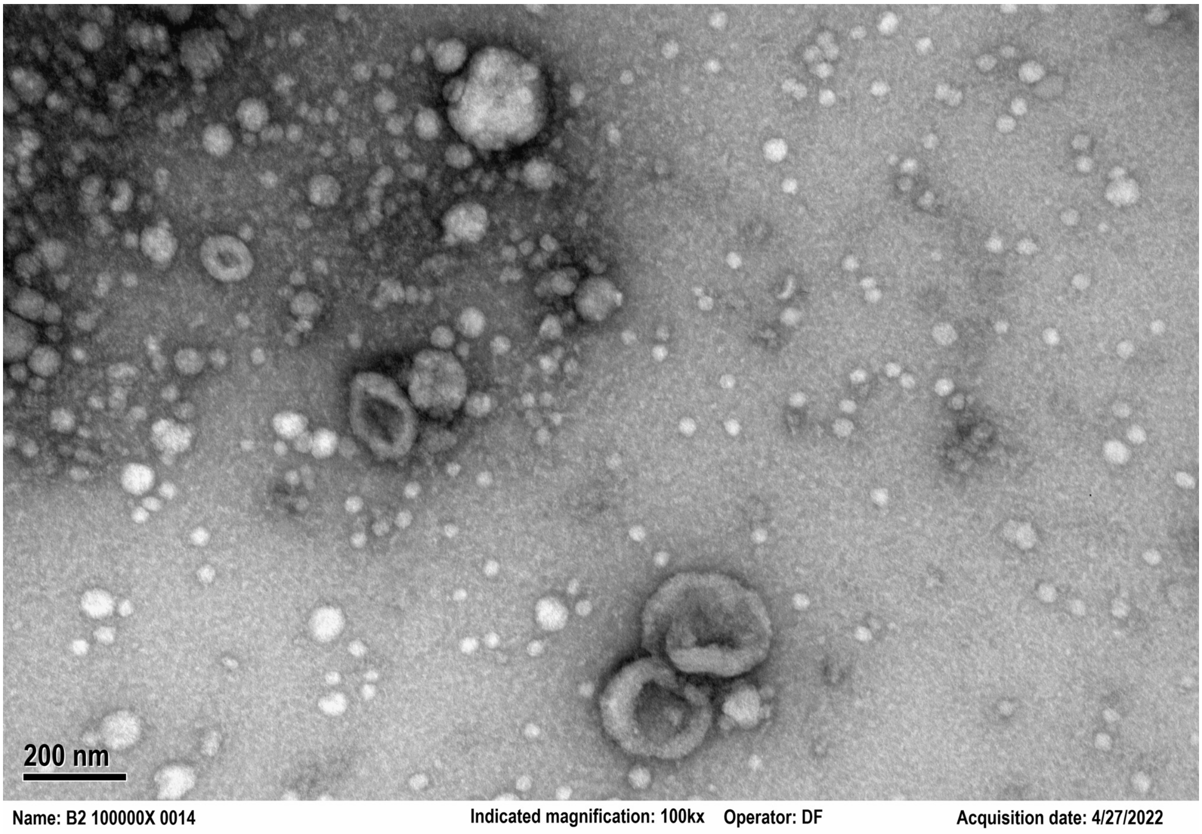

Supplementary Figure S2: Full micrograph from Figure 2B.

Supplementary Figure S3

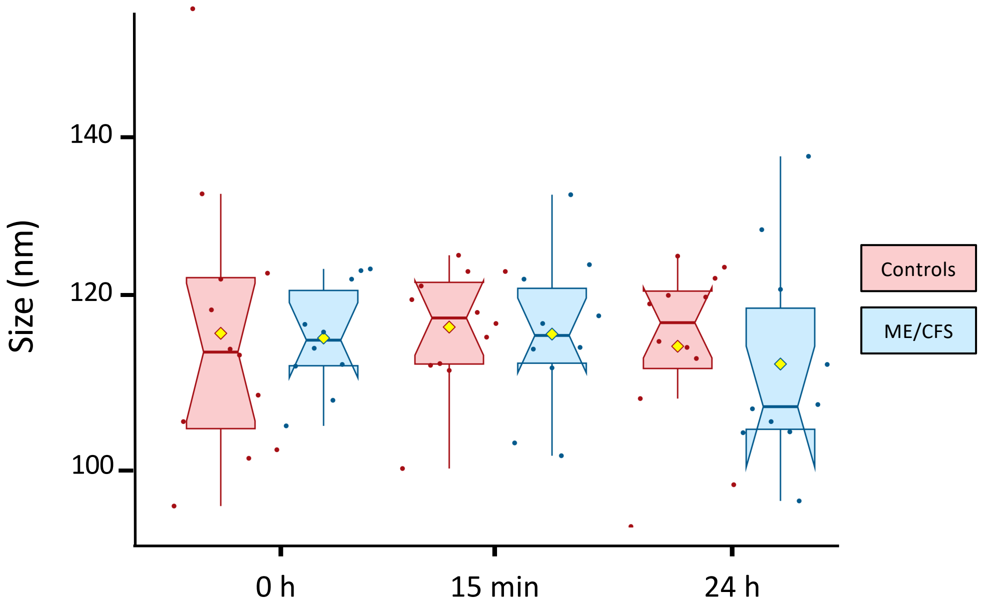

Supplementary Figure S3: Nanoparticle Tracking Analysis. The size of vesicles, measured in nanometers (nm), was analyzed in both ME/CFS subjects and healthy controls. The yellow dot indicates the mean size. The non-parametric Wilcoxon rank sum test was employed to evaluate the significance of differences between the two groups, with a threshold of $p < 0.05$. No significant differences were observed between ME/CFS subjects and healthy controls.

Supplementary Figure S4

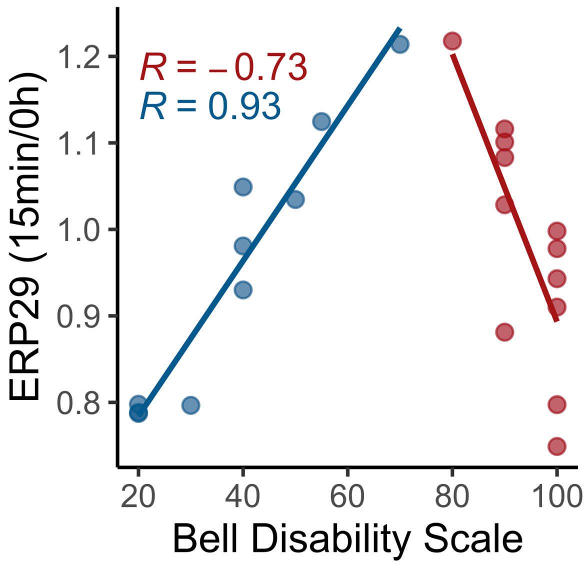

Supplementary Figure S4: Correlation between ERP29 (15min/0h) and Bell Activity Scale scores. Each dot is one subject. The lines are linear regression lines for each group, ME/CFS or control. Spearman's R is shown on each plot for controls (red) and ME/CFS patients (blue). The correlation is significant in ME/CFS patients ($q < 0.1$).
